# Tailored Polymer Hole‐Transporting Materials with Multisite Passivation Functions for Effective Buried‐Interface Engineering of Inverted Quasi‐2D Perovskite Solar Cells

**DOI:** 10.1002/advs.202410807

**Published:** 2024-10-23

**Authors:** Xiujie Zhao, Yinyu Bao, Zhengwu Pan, Qianyu Su, Darui Peng, Deqing Gao, Chengrong Yin, Jianpu Wang, Wei Huang

**Affiliations:** ^1^ Key Laboratory of Flexible Electronics (KLOFE) & Institute of Advanced Materials (IAM) Jiangsu National Synergistic Innovation Center for Advanced Materials (SICAM) Nanjing Tech University (NanjingTech) 30 South Puzhu Road Nanjing 211816 P. R. China; ^2^ Key Laboratory for Organic Electronics and Information Displays & Institute of Advanced Materials (IAM) Nanjing University of Posts & Telecommunications 9 Wenyuan Road Nanjing Jiangsu 210023 P. R. China

**Keywords:** buried‐Interface engineering, inverted quasi‐2D perovskite solar cells, multisite passivation functions, polymer hole‐transporting materials

## Abstract

Although quasi‐2D Ruddlesden‒Popper (RP) perovskite exhibits advantages in stability, their photovoltaic performance are still inferior to 3D counterparts. Optimizing the buried interface of RP perovskite and suppress energetic losses can be a promising approach for enhancing efficiency and stability of inverted quasi‐2D RP perovskite solar cells (PSCs). Among which, constructing polymer hole‐transporting materials (HTMs) with defect passivation functions is of great significance for buried‐interface engineering of inverted quasi‐2D RP PSCs. Herein, by employing side‐chain tailoring strategy to extend the π‐conjugation and regulate functionality of side‐chain groups, target polymer HTMs (PVCz‐ThSMeTPA and PVCz‐ThOMeTPA) with high mobility and multisite passivation functions are achieved. The presence of more sulfur atom‐containing groups in side‐chain endows PVCz‐ThSMeTPA with increased intra/intermolecular interaction, appropriate energy level, and enhanced buried interfacial interactions with quasi‐2D RP perovskite. The hole mobility of PVCz‐ThSMeTPA is up to 9.20 × 10^−4^ cm^2^ V^−1^ S^−1^. Furthermore, PVCz‐ThSMeTPA as multifunctional polymer HTM with multiple chemical anchor sites for buried‐interface engineering of quasi‐2D PSCs can enable effective charge extraction, defects passivation, and perovskite crystallization modulation. Eventually, the PVCz‐ThSMeTPA‐based inverted quasi‐2D PSC achieves a champion power conversion efficiency of 22.37%, which represents one of the highest power conversion efficiencies reported to date for quasi‐2D RP PSCs.

## Graphical abstract

By adopting side‐chain tailoring strategy, two polymer hole‐transporting materials with high mobility and multisite passivation functions are developed for the buried‐interface engineering of inverted quasi‐2D Ruddlesden‒Popper perovskite solar cells (PSCs). Among which, PVCz‐ThSMeTPA‐based inverted quasi‐2D PSCs achieve impressive power conversion efficiency of 22.37% along with excellent thermal and long‐term stability.

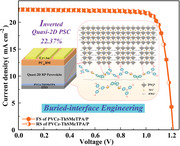

## 1 Introduction

Compared with regular perovskite solar cells (PSCs), the inverted PSCs with the hole‐transporting materials (HTMs) as the underlying substrate of perovskite layer, have the advantages of facile manufacturing process, negligible hysteresis, compatibility with tandem devices, etc.^[^ ^1^, ^2^, ^3^, ^4^, ^5^, ^6^ ^]^ More importantly, the efficiency of the inverted PSCs have made significant progress and approaching those of the regular ones.^[^ ^7^, ^8^, ^9^, ^10^ ^]^ For further commercial development of inverted PSCs, the intrinsic instability problem of 3D perovskites still need to be addressed.^[^ ^11^, ^12^, ^13^ ^]^ Comparing to the 3D perovskites, quasi‐2D Ruddlesden–Popper (RP) perovskites with bulky organic spacer cations exhibit enhanced environmental stability.^[^ ^14^, ^15^, ^16^, ^17^, ^18^ ^]^ Despite better stability, the larger organic spacer cations may enhance the quantum and dielectric constraints of quasi‐2D RP perovskites, which limited the increasement of power conversion efficiencies (PCEs) for quasi‐2D RP PSCs.^[^ ^16^, ^19^, ^20^ ^]^ Up to now, the highest efficiency of inverted PSCs based on quasi‐2D RP perovskites system is just over 21%.^[^ ^15^, ^21^ ^]^ Therefore, it is desirable to further optimize inverted quasi‐2D RP PSCs in order to fulfill both the balance between efficiency and stability.

For achieving highly efficient inverted PSCs, it is desirable to obtain high‐quality perovskite films with low defect density at the grain boundaries and interface which primarily serve as trap states for non‐radiative recombination.^[^ ^22^, ^23^, ^24^ ^]^ In inverted PSCs, the buried interface between the perovskite absorber and HTMs plays a vital role in both hole extraction and perovskite crystallization, significantly affecting the interfacial charge recombination processes.^[^ ^25^, ^26^, ^27^ ^]^ Therefore, effective passivation of defects at the buried interface is crucial for both inverted 3D and quasi‐2D RP PSCs to achieve high performance.^[^ ^28^, ^29^, ^30^, ^31^ ^]^ However, there are still very few reports with effective buried interface modifications in inverted quasi‐2D RP PSCs.

Poly[bis(4‐phenyl)(2,4,6‐trimethylphenyl)amine] (PTAA) and nickel oxide (NiOx) represent the most used HTMs in inverted PSCs still limited by their imperfect interface properties and weak defect passivation ability.^[^ ^32^, ^33^ ^]^ Inserting interfacial buffer layers or passivating materials between perovskite and PTAA/NiOx as the efficient optimization strategies were carried out to improve the surface properties and reduce the buried‐interface defects of perovskites.^[^ ^34^, ^35^, ^36^ ^]^ However, introducing the additional interfacial layer carries the risks of being washed away during subsequent solution preparation process of perovskite film, and meanwhile increases the instability and cost of inverted PSCs.^[^ ^37^ ^]^ To address these issues, polymer HTMs with defect passivation functions that can simultaneously modulate the buried‐interface and crystallinity of perovskites have been explored for inverted 3D PSCs to realize high PCE.^[^ ^38^, ^39^, ^40^, ^41^ ^]^ More importantly, this strategy is also applicable to the performance improvements of inverted quasi‐2D RP PSCs.

Based on this, two novel side‐chain polymer HTMs (PVCz‐ThSMeTPA and PVCz‐ThOMeTPA) with multiple functional groups were developed for the buried interface engineering of inverted quasi‐2D RP PSCs. In our design of polymer HTMs, the arrangement of side‐chain groups with enhanced structural planarity and extended π‐conjugation are restricted by the flexible non‐conjugated polyvinyl backbone, which favor excellent molecular π−π interactions. Meanwhile, the sulfur atom‐containing thienyl and methylthio groups in PVCz‐ThSMeTPA may enhance good intra‐ and intermolecular packing via S···S interactions, which are beneficial to charge transport, and leading to high carrier mobility of PVCz‐ThSMeTPA up to 9.20 × 10^−4^ cm^2^ V^−1^ S^−1^. The two novel side‐chain polymer HTMs also exhibit desirable energy level alignment with the valence band maximum (VBM) of quasi‐2D RP perovskite, which facilitates efficient carrier transport at buried interface. PVCz‐ThSMeTPA exhibits a deeper highest occupied molecular orbital (HOMO) level than PVCz‐ThOMeTPA owing to the π‐acceptor capability of methylthio groups. In addition, the sulfur atoms in thienyl and methylthio groups of PVCz‐ThSMeTPA and PVCz‐ThOMeTPA are intended to control crystal formation and passivate buried interface defects by coordinating with Pb^2+^ in the quasi‐2D RP perovskite film. It was also found that PVCz‐ThSMeTPA with multiple chemical anchor sites exhibit enhanced defect passivation ability on the defect of quasi‐2D RP perovskite compared to PVCz‐ThOMeTPA and PTAA. As result, the inverted quasi‐2D RP PSCs using PVCz‐ThSMeTPA as HTMs obtained the champion PCE of 22.37%, which outperforms the corresponding PCE of PVCz‐ThOMeTPA and PTAA‐based devices. More importantly, this PCE is among the top reported values for inverted quasi‐2D RP PSCs.

## 2 Results and Discussion

**Figure 1:**
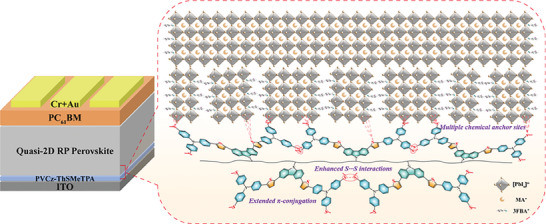
Schematic illustration of molecular characteristic of PVCz‐ThSMeTPA, and buried‐interface engineering of quasi‐2D RP perovskite by the PVCz‐ThSMeTPA.

**Figure 2:**
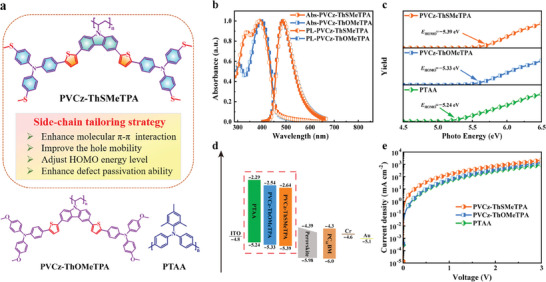
a) Chemical structures of PVCz‐ThSMeTPA, PVCz‐ThOMeTPA and PTAA. b) Normalized UV–vis absorption‐PL emission spectra of PVCz‐ThSMeTPA and PVCz‐ThOMeTPA films. c) PYS of PVCz‐ThSMeTPA and PVCz‐ThOMeTPA. d) Structure and energy level diagram of functional layers in inverted PSCs. e) Hole‐injection characteristics measured by the SCLC method based on the device structure of ITO/PEDOT: PSS/HTMs/MoO_3_/Au.

**Figure** **1** displays the polymer structures of PVCz‐ThSMeTPA and the mechanisms of defect passivation and crystallization regulation of perovskite films by PVCz‐ThSMeTPA. Regarding the molecular design strategy of PVCz‐ThSMeTPA (**Figure** **2**a), based on flexible non‐conjugated polyvinyl backbone, we used side‐chain tailoring strategy to introduce triphenylaminothiophene groups into side‐chain units in order to increase intra/intermolecular π−π packing interactions via extended π‐conjugation and S···S interactions. Furthermore, the methylthio moieties were employed in the structure design of PVCz‐ThSMeTPA to improve its defect passivation ability on quasi‐2D RP perovskites, and PVCz‐ThOMeTPA with methoxyl moieties was designed for comparison. The stronger coordination ability between sulfur atom and Pb^2+^ means that PVCz‐ThSMeTPA can attract the undercoordinated Pb^2+^ in the perovskite layer, reducing lead clusters and the defects in the perovskite buried interface.

PVCz‐ThSMeTPA and PVCz‐ThOMeTPA were synthesized via radical polymerization, and triphenylaminothiophene‐functionalized vinylcarbazole monomers were obtained from Suzuki coupling reactions. The synthetic routes of two polymers are shown in Figures S1‐S2 (Supporting Information). The structures of the intermediate monomers were characterized by ^1^HNMR and ^13^CNMR (Figures S11–30, Supporting Information). The synthetic costs of PVCz‐ThSMeTPA and PVCz‐ThOMeTPA were estimated to be low at $12.4 and 8.5 g^−1^, providing substantial cost compared to PTAA ($423.3 g^−1^) (detailed cost analysis, Tables S1 and S2, Supporting Information). PVCz‐ThSMeTPA and PVCz‐ThOMeTPA were obtained with a number‐average molecular weight (*M*_n_) of 25 819 and 19 304, as well as polydispersity index (PDI) of 2.04 and 3.29 for them, respectively.

The optical properties of PVCz‐ThSMeTPA and PVCz‐ThOMeTPA were characterized and analyzed by UV–vis absorption and photoluminescence (PL) spectroscopy to help clarify the charge transfer mechanism of the materials. Figure 2b shows the normalized absorption and PL spectra of polymer PVCz‐ThSMeTPA and PVCz‐ThOMeTPA thin films. PVCz‐ ThSMeTPA and PVCz‐ThOMeTPA have almost identical absorption bands in the UV–vis region with the peak maximum (*λ*_abs, max_) located at 390 and 396 nm, respectively, which is attributed to the π–π* transition between the conjugated side‐chain units. The maximum emission peak of PVCz‐ThSMeTPA and PVCz‐ThOMeTPA in PL spectrum is 487 and 486 nm respectively. Compared with the solution state spectra (Figure S3, Supporting Information), PVCz‐ThSMeTPA and PVCz‐ThOMeTPA films show significant red‐shifts due to the effective π–π stacking interaction of polymers in solid state. According to the ultraviolet absorption spectrum, the optical bandgaps (*E*_g_) of PVCz‐ThSMeTPA and PVCz‐ThOMeTPA are 2.75 and 2.79 eV, respectively. In order to determine the HOMO levels of PVCz‐ThSMeTPA and PVCz‐ThOMeTPA, we first used the IPS‐4 ionization energy measurement system to conduct photoelectron spectroscopy analysis of PVCz‐ThSMeTPA and PVCz‐ThOMeTPA in N_2_ atmosphere, as shown in Figure 2c. The HOMO levels of PTAA, PVCz‐ThSMeTPA, and PVCz‐ThOMeTPA were measured to be −5.24, −5.39 and −5.33 eV, respectively. The HOMO levels of PVCz‐ThSMeTPA and PVCz‐ThOMeTPA matched the valence band (−5.98 eV) of our perovskite more closely than that of PTAA (Figure 2d). Notably, due to the π‐acceptor capability of the sulfur atom, PVCz‐ThSMeTPA showed a downshifted HOMO level relative to PVCz‐ThOMeTPA, which is favorable for hole extraction and reducing the *V*_oc_ losses. And the LUMO levels of PVCz‐ThSMeTPA and PVCz‐ThOMeTPA were calculated to be −2.64 and −2.54 eV by the sum of the *E*_g_ and the HOMO. Cyclic voltammetry (CV) measurements were also carried out to estimate the HOMO energy levels of PVCz‐ThSMeTPA and PVCz‐ThOMeTPA, as shown in Figure S5 (Supporting Information). The HOMO and LUMO levels obtained by the two measurement methods were basically the same.

The space charge limited current (SCLC) method was used to estimate the hole mobility of PVCz‐ThSMeTPA and PVCz‐ThOMeTPA (Figure 2e). The hole only devices with structure of indium tin oxide (ITO)/poly (3,4‐ethylenedioxythiophene): Polystyrene sulfonate (PEDOT: PSS)/polymer HTM/MoO_3_/Ag were fabricated. The hole mobilities of PVCz‐ThSMeTPA and PVCz‐ThOMeTPA are determined to be 9.20 × 10^−4^ and 5.72 × 10^−4^ cm^2^ V^−1^ S^−1^, respectively, higher than that of PTAA (3.71 × 10^−4^ cm^2^ V^−1^ S^−1^). Compared with our previous side‐chain polymer HTMs and PTAA, the highest hole mobility of PVCz‐ThSMeTPA can be attributed to its side‐chain groups with enhanced structural planarity and extended π‐conjugation as well as increased S···S interactions, which favor excellent intra‐ and intermolecular π–π packing interactions.^[^ ^18^, ^42^, ^43^ ^]^ Thermogravimetric analysis (TGA) and differential scanning calorimetry (DSC) were used to characterize and analyze the thermal stability of PVCz‐ThSMeTPA and PVCz‐ThOMeTPA. As shown in Figure S6 (Supporting Information), the degradation temperatures (*T*_d_) with 5% weight loss of PVCz‐ThSMeTPA and PVCz‐ThOMeTPA are 423 and 451 °C, respectively. And both polymers did not exhibit significant glass transition and melting peaks. The results reflect good thermal stability of two polymers.

**Table** **1** summarizes detailed data on the optical, thermodynamic, electrochemical, and photoelectric properties of PVCz‐ThSMeTPA and PVCz‐ThOMeTPA.

**Table 1:** The optical, thermodynamic, electrochemical and photoelectrical properties of PVCz‐ThSMeTPA and PVCz‐ThOMeTPA.

HTMs	*λ*_abs_ [nm]	*λ*_emi_ [nm]	*T*_d_ (°C)	*E*_g_ (eV)^a)^	*E*_HOMO_ (eV)^b)^	*E*_HOMO_ (eV)^c)^	*E*_LUMO_ (eV)^d)^	*M*_n_ */M*_w_	*µ*_h_ [cm^2^ V^‒1^ s^‒1^] ^e)^
PVCz‐ThSMeTPA	390	487	422	2.75	‒5.39	‒5.40	‒2.64	25819/52646	9.20 × 10^−4^
PVCz‐ThOMeTPA	396	486	451	2.79	‒5.33	‒5.34	‒2.54	19304/63494	5.72 × 10^−4^

^a)^ *E*_g_ obtained from the edge of absorption;

^b)^ Determined by IPS‐4 ionization energy measurement system;

^c)^ Determined first oxidation potential from film‐based CV;

^d)^ LUMO calculated by LUMO = HOMO + *E*_g_;

^e)^ Hole mobility estimated from SCLC.

**Figure 3:**
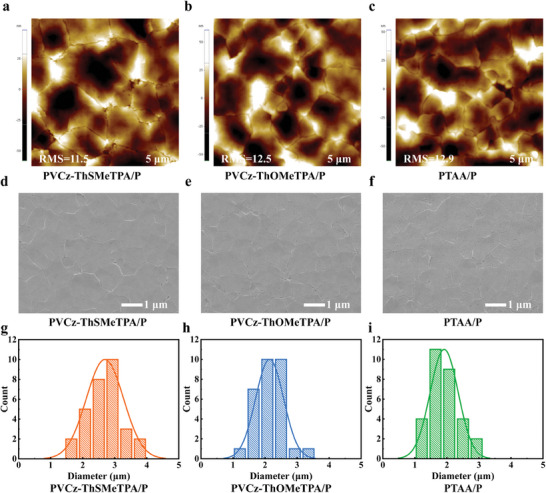
a‐c) AFM images of perovskite films on PVCz‐ThSMeTPA, PVCz‐ThOMeTPA, and PTAA. d–f) SEM images of the perovskite films on different polymer HTMs. g–i) The corresponding grain size distributions of perovskites on the different HTMs.

The crystal morphology and wettability of the quasi‐2D RP perovskites deposited on PVCz‐ThSMeTPA, PVCz‐ThOMeTPA and PTAA are shown in **Figure** **3**. The film morphology of polymer HTMs will affect the growth of quasi‐2D RP perovskite in the inverted PSCs. To better understand how this works, we conducted the following tests. Firstly, the ITO substrates coated only with HTMs were characterized by atomic force microscopy (AFM), as shown in Figure S7 (Supporting Information). The ITO substrates coated with polymer HTMs all show uniform film morphology. However, ITO substrates coated with PVCz‐ThSMeTPA and PVCz‐ThOMeTPA showed relatively lower root‐mean‐square (RMS) roughness compared to PTAA. In addition, in the dimethylformamide (DMF) contact Angle test, PVCz‐ThSMeTPA and PVCz‐ThOMeTPA both have small DMF contact angles, which are 6.1° and 12.5° respectively, as shown in Figure S8 (Supporting Information). These results indicate that PVCz‐ThSMeTPA and PVCz‐ThOMeTPA as HTM substrates are beneficial for the spreading and growth of quasi‐2D RP perovskite, thereby obtaining dense, uniform, and smooth perovskite films. We further studied the crystallization growth of quasi‐2D RP perovskite films on different polymer HTMs using AFM, as shown in Figure 3a–c. The quasi‐2D RP perovskite films based on PVCz‐ThSMeTPA and PVCz‐ThOMeTPA exhibit lower RMS roughness at 11.5 and 12.5 nm, respectively, while the films based on PTAA reached 12.9 nm. Secondly, the study on the crystallization growth of perovskite films was deepened through top‐view SEM, as shown in Figure 3d–f, and the corresponding grain size distributions are illustrated in the Figure 3g–i. The quasi‐2D RP perovskite film deposited on PVCz‐ThSMeTPA shows fewest pinholes and largest grain size, as compared with PVCz‐ThSMeTPA and PTAA based perovskite films. These results further demonstrate that PVCz‐ThSMeTPA and PVCz‐ThOMeTPA are beneficial for the crystal growth and defect passivation of quasi‐2D RP perovskite films, thereby reducing non‐radiative recombination and improving performance of inverted PSCs.

**Figure 4:**
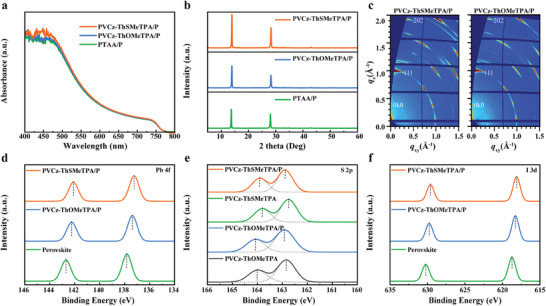
a) UV–vis absorption spectra of perovskite films deposited on PVCz‐ThSMeTPA, PVCz‐ThOMeTPA, and PTAA. b) XRD patterns of perovskite films deposited on three polymers. c) GIWAX patterns of PVCz‐ThSMeTPA and PVCz‐ThOMeTPA based perovskite films. XPS spectra of d) Pb 4*f*, e) S 2*p* and f) I 3*d* levels in the perovskite film, pristine polymer HTMs, and the bilayered perovskite/polymer HTMs films.

We studied the effects of three HTMs on the absorbance of perovskite layers, as shown in **Figure** **4**a. Compared with PTAA, the light absorption of perovskite films deposited on PVCz‐ThSMeTPA and PVCz‐ThOMeTPA was enhanced, which is due to the increase in grain size of perovskite. As shown in Figure 4b and X‐ray diffraction (XRD) spectra measurements were carried to investigate the crystallization and morphology of quasi‐2D RP perovskite films deposited on different polymer HTMs. All HTM/perovskite films display the same diffraction peaks at 14.14° and 28.50°. The peak intensity of perovskite film grown on PVCz‐ThSMeTPA was much stronger than that of PTAA and PVCz‐ThOMeTPA, which indicates PVCz‐ThSMeTPA as underlaying HTMs can enhance the crystallinity of perovskite films. The crystal orientation of the 2D RP perovskites based on PVCz‐ThSMeTPA and PVCz‐ThOMeTPA were further confirmed by using the GIWAXS measurements. As shown in Figure 2c, the quasi‐2D RP perovskite films exhibited sharp and discrete Bragg spots, suggesting their excellent crystallinity and well‐aligned perovskite structure. The strong diffraction peaks of (111) and (202) along the *q*_xy_ and *q*_z_ direction suggest that the vertical orientation of 2D perovskite crystals on our polymer HTMs, which could promotes effective charge transport between the front and back electrodes.^[^ ^17^, ^44^ ^]^ In particular, the formation of 2D RP perovskite was further revealed by the appearance of the (0k0) diffraction peak at *q*_z_ ≈ 0.35 Å^−1^. X‐ray photoelectron spectroscopy (XPS) was used to probe the coordination interaction between perovskite and polymer HTM. As illustrated in Figure 4d, there are two peaks at 137.83 and 142.69 eV determined as Pb 4*f*_7/2_ and Pb 4*f*_5/2_ in bare quasi‐2D RP perovskite film, respectively. As compared, the Pb 4*f* peaks of the quasi‐2D RP perovskites with PVCz‐ThSMeTPA and PVCz‐ThOMeTPA all show shift to the lower binding energy, indicating the interaction between quasi‐2D RP perovskite layer and polymer HTMs. As shown in Figure 4e, two characteristic peaks ascribed to S 2*p*_1/2_ and S 2*p*_3/2_ were observed at 162.74 and 163.80 for PVCz‐ThSMeTPA, 162.83 and 164.01 for PVCz‐ThOMeTPA, while perovskite/PVCz‐ThSMeTPA and perovskite/PVCz‐ThOMeTPA bilayers reveal the peaks of S 2*p* shift to higher binding energy. The above Pb 4*f* and S 2*p* peak shift in the XPS spectra confirms the passivation effect of PVCz‐ThSMeTPA and PVCz‐ThOMeTPA on the buried interface of quasi‐2D RP perovskite. More importantly, quasi‐2D RP perovskite/PVCz‐ThSMeTPA bilayer exhibits a larger shift in main peaks for Pb 4*f* and S 2*p* orbits compared with that of the quasi‐2D RP perovskite/PVCz‐ThOMeTPA bilayer, which demonstrates that more favorable interaction of Pb^2+^ ions with methylthio groups in PVCz‐ThSMeTPA compared to methoxyl groups in PVCz‐ThOMeTPA. Furthermore, the characteristic peak of I 3*d* in the PVCz‐ThSMeTPA based perovskite film also has an obvious shift (Figure 4f), confirming the interactions between the PVCz‐ThSMeTPA and perovskite.

**Figure 5:**
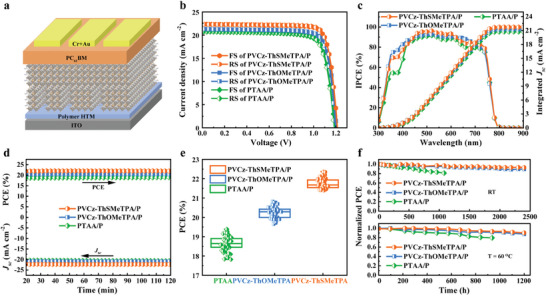
a) The device architecture of inverted quasi‐2D RP PSC. b) *J*–*V* curve based on forward scan and reverse scan of three HTMs champion cells recorded under AM1.5G (100 mW cm^‒2^). c) IPCE spectra and integrated short circuit current density of three HTMs‐based PSCs. d) Steady‐state output of the maximum power point for devices based on three polymer HTMs. e) Histogram of efficiency distribution of three polymer HTMs based PSCs. f) Stability tests of three polymer HTMs based inverted quasi‐2D PSCs kept in inert atmosphere for long‐term stability and heat stability under 60 °C continuous annealing.

In order to further evaluate the impact of PVCz‐ThSMeTPA and PVCz‐ThOMeTPA as HTMs on the photovoltaic performance of inverted quasi‐2D RP PSCs, device structures of ITO/polymer HTM/quasi‐2D RP perovskite/PC_61_BM/Cr/Au were fabricated (**Figure** **5**a). The perovskite absorber layer was composed of (3FBA)_2_MA_3_Pb_4_I_13_ (3FBA = 3‐ fluorobenzylammonium, MA = methylammonium), PC_61_BM is used as electron‐transporting material (ETM), Cr and Au acts as a charge barrier layer and cathode, respectively. Figure 5b shows the current‐voltage (*J‒V*) characteristic curves of the optimal PSCs deposited on different polymer HTMs after forward and reverse scanning, and detailed data is summarized in **Table** **2**. The reference PTAA‐based quasi‐2D RP PSCs showed a maximum PCE of 19.60% in a reverse scan with a short‐circuit current density (*J*_sc_) of 20.86 mA cm^−2^, open‐circuitvoltage (*V*_oc_) of 1.18 V, and fill factor (FF) of 0.79. The inverted quasi‐2D RP PSCs based on PVCz‐ThOMeTPA exhibited slightly higher PCE of 20.82% than the PTAA based PSCs. The PVCz‐ThSMeTPA based inverted quasi‐2D RP PSC exhibits the best performance with PCE of 22.37%, with *J*_sc_ of 22.42 mA cm^−2^, *V*_oc_ of 1.22 V and FF of 0.82, respectively, which is one of the best results for inverted quasi‐2D RP PSCs. The excellent performance of PVCz‐ThSMeTPA based PSCs mainly attributed to the improved HOMO energy level and charge mobility as well as defect passivation ability of PVCz‐ThSMeTPA. The PSCs based on PVCz‐ThSMeTPA and PVCz‐ThOMeTPA both show low hysteresis effect. Incident photon‐to‐electron conversion efficiency (IPCE) further testified the reliability of the *J*_sc_ measured by the *J‒V* in Figure 5c. As shown in Figure 5c, PVCz‐ThSMeTPA and PVCz‐ThOMeTPA based devices all show higher photo‐response in a broad spectral range between 300 and 800 nm. Note that the significantly dropped IPCE at lower wavelengths for PTAA based PSCs could be attributed to its dominated low n‐value phases (n = 2 and 3) in perovskite, while the photogenerated exciton in n = 2 phase couldn't be separated to free charge carrier and transport efficiently. The integrated *J*_sc_ of PVCz‐ThSMeTPA, PVCz‐ThOMeTPA and PTAA based quasi‐2D RP PSCs are 21.73, 21.27 and 20.77 mA cm^−2^, respectively, which are within 5% of the *J*_sc_ errors obtained in the *J‒V* curve. Subsequently, the steady‐state photocurrent of the devices was further recorded to illustrate the stability of the output efficiency, as shown in Figure 5d. Under continuous irradiation for 120 min, the devices with PVCz‐ThSMeTPA, PVCz‐ThOMeTPA and PTAA all show stabilized the output photocurrent at the maximum power point, indicating that the efficiency output is stable. In addition, under the same test conditions, we selected 50 devices from each polymer HTM for PCE distribution analysis, as shown in Figure 5e and Figure S9 (Supporting Information). Compared to PTAA based quasi‐2D RP PSCs, the devices with PVCz‐ThSMeTPA and PVCz‐ThOMeTPA have narrower PCE distribution, indicating their excellent reproducibility.

**Table 2:** Photovoltaic metrics of champion devices based on PVCz‐ThSMeTPA, PVCz‐ThOMeTPA and PTAA.

HTL	Scan directions	*V*_oc_[V]	*J*_sc_[mA cm^‒2^]	FF	PCE [%]
PVCz‐ThSMeTPA	Forward	1.22	22.48	0.81	22.12
	Reverse	1.22	22.42	0.82	22.37
PVCz‐ThOMeTPA	Forward	1.20	21.62	0.79	20.63
	Reverse	1.20	21.37	0.81	20.82
PTAA	Forward	1.18	21.24	0.77	19.39
	Reverse	1.18	20.86	0.79	19.60

The stability of the quasi‐2D RP PSCs with different HTMs were investigated. As shown in Figure 5f, the unencapsulated quasi‐2D RP PSCs with PVCz‐ThSMeTPA and PVCz‐ThOMeTPA retained 92.9% and 89.4% of its initial efficiency when stored in inert environment for 2400 h. Nevertheless, the PCE of PTAA based device dropped quickly, which is mainly due to the intrinsic bulk and interface defect induced irreversible degradation in the perovskite. According to the trend of PVCz‐ThsMeTPA and PVCz‐ThOMeTPA modified device in this test of stability, they all show PCE recovery with different rate in the period of 250 h to 400 h. The PCE improvement under illumination after storage in the dark is commonly attributed to the neutralization of interfacial defects by photogenerated charge carriers or to changes in the built‐in electric field due to ion migration.^[^ ^45^, ^46^ ^]^ Furthermore, due to the higher hole mobility and better defect passivation ability of PVCz‐ThSMeTPA than PVCz‐ThOMeTPA, PVCz‐ThsMeTPA modified device can effectively reduce charge accumulation and decrease the defect at the interface of perovskite, thus reducing ion migration under light. As result, the PCE of PVCz‐ThSMeTPA modified device recovered better than PVCz‐ThOMeTPA based device in the period of 250 h to 400 h. Moreover, after continuous annealing at 60 °C for 1200 h, the unencapsulated devices with PVCz‐ThSMeTPA and PVCz‐ThOMeTPA still maintained initial efficiency of 90.2% and 87.6%, while the unencapsulated devices with PTAA dropped to 79.5% after just over 936 hours. The stability measurement results suggest that the buried‐interface engineering using PVCz‐ThSMeTPA is an effective approach to significantly enhance efficiency and operational reliability of inverted quasi‐2D RP PSCs.

**Figure 6:**
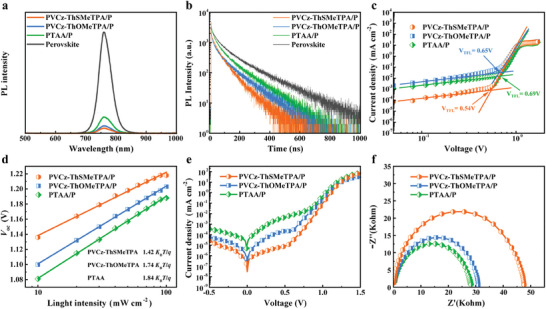
a) Steady‐state PL spectra and b) Time‐resolved PL of PVCz‐ThSMeTPA/perovskite, PVCz‐ThOMeTPA/perovskite, PTAA/perovskite and original perovskite films. c) *J‒V* curves of the hole only devices. d) Light dependence of *V*_oc_ in PVCz‐ThSMeTPA, PVCz‐ThOMeTPA and PTAA based PSCs. e) Dark current of PVCz‐ThSMeTPA, PVCz‐ThOMeTPA and PTAA based PSCs. f) Electrochemical impedance spectroscopy of the PSCs with three polymer HTMs.

To analyze the effect of different polymer HTMs on carrier transport in perovskite films, steady‐state PL and time‐resolved PL (TRPL) measurements were recorded in **Figure** **6**a,b. The device with PVCz‐ThSMeTPA exhibits a much lower PL intensity compared to PTAA and PVCz‐ThOMeTPA, meaning more efficient hole extraction at perovskite/HTL interfaces. This was further confirmed by TRPL which presented shorter fluorescence quenching lifetime for the devices with PVCz‐ThSMeTPA than PTAA and PVCz‐ThOMeTPA, indicating faster hole extraction (Figure 6b; Table S3, Supporting Information). To further assess the effect of PVCz‐ThSMeTPA and PVCz‐ThOMeTPA on the defect density of quasi‐2D RP perovskite, SCLC experiments were carried out. Figure 6c shows the *J‒V* curves of the pure hole devices with ITO/PEDOT: PSS/polymer HTM/perovskite/PTAA/Ag structure. The defect density calculation formula (1) for perovskite thin films is as follows:

(1) $$N_{\mathrm{defects}}=\frac{2\varepsilon \varepsilon_{0}V_{\mathrm{TFL}}}{qL^{2}}\;$$

where *ε* and *ε*_0_ are the relative dielectric constant and vacuum dielectric constant, respectively, *L* is the thickness of the perovskite film, *q* is the elementary charge, and *V*_TFL_ is the trap‐filled limit voltage. The *N*_defects_ for devices with PVCz‐ThSMeTPA, PVCz‐ThOMeTPA and PTAA were calculated to be 3.11 × 10^15^, 4.88 × 10^15^ and 5.71 × 10^15^ cm^−3^. The decreased trap density of the PVCz‐ThSMeTPA based quasi‐2D RP PSCs were attributed to its enhanced carrier mobility and more efficient passivation ability, which could reduce the trap‐assisted recombination and facilitate the efficient charge transport. Furthermore, according to the linear relationship between *V*_oc_ and light intensity of the devices in Figure 6d, the fitted slope of dependence decreases to 1.42 *K*_B_ *T*/*q* for device with PVCz‐ThSMeTPA, compared with 1.74 and 1.84 *K*_B_ *T*/*q* for device with PVCz‐ThOMeTPA and PTAA, where *K*_B_, *T* and *q* represent the Boltzmann constant, temperature and elementary charge, respectively. The results demonstrate less trap‐assisted recombination in PVCz‐ThSMeTPA based quasi‐2D RP PSCs.

In addition, the dark‐current measurements were conducted to further elucidate the charge recombination kinetics in our PSCs, as shown in Figure 6e. The *R*_sh_ which is the shunt resistance of the devices with PVCz‐ThSMeTPA and PVCz‐ThOMeTPA are significantly higher than that of the devices with PTAA, this means that the devices with PVCz‐ThSMeTPA and PVCz‐ThOMeTPA have a smaller leakage current. It also means that their perovskite films are of higher quality and less non‐radiative recombination. According to the Shockley equation, we can calculate *V*_oc_ from Equation (2):

(2) $$V_{\mathrm{oc}}=\frac{nK_{B}T}{q}\;ln\left(\frac{J_{sc}}{J_{0}}\right)\;$$

where *n* is the ideality factor, *K*_B_ is the Boltzmann constant, *T* is the absolute temperature, *q* is the elementary charge, *J*_sc_ is the photogenerated short‐circuit current density, and *J*_0_ is the reverse saturation current density. Lower *J*_0_ means higher *V*_oc_. The smaller *J*_0_ of the devices with PVCz‐ThSMeTPA (*J*_0_ between 10^−15^ and 10^−16 ^mA cm^−2^) and PVCz‐ThOMeTPA (*J*_0_ between 10^−12^ and 10^−13 ^mA cm^−2^) means that they have a higher *V*_oc_ compared to PTAA (*J*_0_ between 10^−11^ and 10^−12 ^mA cm^−2^), which is also consistent with the photovoltaic performance of the devices in Table 2. Moreover, electrochemical impedance spectroscopy (EIS) was used to analyze the charge recombination kinetics of the devices, as shown in Figure 6f. The devices with PVCz‐ThSMeTPA showed much larger recombination resistance (*R*_rec_) compared with others, indicating that PVCz‐ThSMeTPA can effectively suppress charge recombination.

## 3 Conclusion

In summary, we adopt side‐chain tailoring strategy to obtain two polymer HTMs (PVCz‐ThSMeTPA and PVCz‐ThOMeTPA) with high mobility and multisite passivation functions for the buried interface engineering of inverted quasi‐2D RP PSCs. The extended π‐conjugation and increased S···S interactions in the side‐chain of two polymer HTMs could enhance intra/intermolecular π‐π interaction, leading to high mobility of 9.20 × 10^−4^ cm^2^ V^−1^ S^−1^ for PVCz‐ThSMeTPA. Additionally, the sulfur atom‐containing thienyl and methylthio groups in PVCz‐ThSMeTPA and PVCz‐ThOMeTPA are intended to passivate the defects at the buried interface of quasi‐2D RP perovskite by coordinating with Pb^2+^ ions. As expected, we also confirmed that PVCz‐ThSMeTPA with multiple chemical anchor sites exhibit enhanced defect passivation and crystallization modulation ability on quasi‐2D RP perovskite compared to PVCz‐ThOMeTPA and PTAA, which could reduce charge recombination losses efficiently. Consequently, the inverted quasi‐2D RP PSCs incorporating the PVCz‐ThSMeTPA as HTM achieved a champion PCE of 22.37%, along with excellent thermal and long‐term stability.

## Conflict of Interest

The authors declare no conflict of interest.

## Supporting information



Supporting Information

## Data Availability

The data that support the findings of this study are available in the supplementary material of this article.
