# Supplementary material for: Tailored Polymer Hole‐Transporting Materials with Multisite Passivation Functions for Effective Buried‐Interface Engineering of Inverted Quasi‐2D Perovskite Solar Cells
Source: Adv Sci (Weinh). 2024 Oct 23;11(46):2410807. doi: 10.1002/advs.202410807 (PMC11633489; doi:10.1002/advs.202410807)
Supplement: Supplementary file 1 — Supporting Information [file ADVS-11-2410807-s001.docx]

Supporting Information

**Tailored Polymer Hole-transporting Materials With Multisite Passivation Functions** **For Effective Buried-Interface Engineering of Inverted quasi-2D Perovskite Solar Cells**

Xiujie Zhao, Yinyu Bao, Zhengwu Pan, Qianyu Su, Darui Peng, Deqing Gao, Chengrong Yin,* Jianpu Wang, Wei Huang

**Experimental Section**

*Materials and soluntions*: 4-Iodoanisole, 4-Iodothioanisole, 4-Bromoaniline, 1,10-Phenanthroline, 2-Thiopheneboronic acid, Pd(PPh_3_)_4_, K_2_CO_3_, 2-isopropoxy-4,4,5,5-tetramethyl-1,3,2-dioxaborolane, 3,6-Diiodo-9H-carbazole, n-Butyllithium, KOH, Toluene, THF, Hydroquinone, IPA, Methanol, N-hexane, DMF (Alfa Aesar, 99.8%), CB (Alfa Aesar, 99.8%), PTAA (p-OLED), PC_61_BM (p-OLED), PbI_2_ (TCI), Aurum (AU, 99.99%) and other reagents were used as received from commercial sources and used without further purification. All the solvents were treated according to the standard procedures.

*Synthesis of 3FBAI*: 3-Fluorobenzylamine (1 mmol) was dissolved in ethanol solvent (20 ml), pretreated at 0 °C for 10min, hydroiodic acid (1 mmol, 57%) was added to the reaction and stirred for 2 h to synthesize 3FBAI. After that, the reaction solvent was spin-dried, dissolved in a trace amount of ethanol, and then washed with anhydrous ether and Petroleum ether. Repeated 3-4 times to remove the residual hydroiodic acid from the solvent reaction, and then the obtained product was dried under vacuum at 60 °C for 12 h. (Hydroiodic acid is Aladdin 56%–58%; 3-Fluorobenzylamine is Energy Chemical).

*Preparation of quasi-2D perovskite precursor solution:* The perovskite precursor solution (30wt%, molar ratio 2.2: 3.5: 4 for 3FBAI/MACl/PbI_2_) was prepared in DMF and stirred overnight in a N_2_ glovebox."

*Devices Fabrication*: The indium tin oxide (ITO)-coated glass substrate (1.0 ×1.0 cm) was washed with acetone and ethanol in turn, and then subjected to plasma treatment for 15 min. Transfer the substrate to a nitrogen glove box. The chlorobenzene solution of PVCz-ThSMeTPA, PVCz-ThOMeTPA or PTAA was spin-coated on the ITO substrate, the obtained film was annealed at 90 °C for 10 min. Preheating at 140 °C for 3 min, coating the perovskite precursor solution on HTM at 5000 rpm Spin coating for 20 s, and then annealing at 90 °C for 13 min. Then PC_61_BM solution (20 mg/ml) was coated on the membrane at 1000 rpm Spin coating for 40 s. Then separate the chromium and gold at Evaporate under a vacuum of 6×10^−5^ Pa. The active area of the perovskite solar cells prepared is 0.03 cm^2^.

*Synthesis of Br-SMeTPA*: To a solution of 4-Iodothioanisole (1.25 g, 5 mmol) and 4-bromoaniline (0.86 g, 5.2 mmol) in 60 mL toluene, 1,10-phenanthroline (0.018 g, 0.1 mmol), CuCl (0.001 g, 0.1 mmol) and KOH (1.12 g, 20 mmol) were added in a 250 mL Schlenk flask. The mixture was heated to 135℃ for 48 hours under nitrogen. After cooling to RT, the reaction mixture was extracted with DCM. The combined organic layer was dried over anhydrous MgSO_4_. After the solvent was evaporated, the residue was purified using column chromatography (silica gel, PE: DCM = 1: 1) to obtain product as Br-SMeTPA (1.74 g, 83.5%). ^1^H NMR (400 MHz, Chloroform-d) δ(ppm): 7.33‒7.30 (m, 2H), 7.22‒7.14 (m, 4H), 7.03‒6.98 (m, 4H), 6.96‒6.88 (m, 2H), 2.47 (s, 6H). ^13^C NMR (101 MHz, Chloroform-d) δ(ppm): 146.71, 144.86, 132.49, 132.29, 128.53, 124.89, 114.93, 16.80.

*Synthesis of ThSMeTPA*: Br-SMeTPA (0.416 g, 1 mmol) was placed in a 250 mL Schlenk flask together with 2-thiopheneboronic acid (0.130 g, 1.1 mmol), Pd (PPh_3_)_4_ (0.046 g, 0.02 mmol) and K_2_CO_3_ (0.28 g, 5 mmol). The system was cycled three times with N_2_, then H_2_O and DMF (100 mL) was added. The reaction was heated under nitrogen for 12 hours at 110 ℃. After cooling to RT, the reaction mixture was extracted with DCM and H_2_O. The combined organic layer was dried over anhydrous MgSO_4_. After the solvent was evaporated, the residue was purified using column chromatography (silica gel, PE: DCM = 5: 1) to obtain product as ThSMeTPA (0.339 g, 80.8%). ^1^H NMR (400 MHz, DMSO-d6) δ(ppm): 7.63‒7.55 (m, 2H), 7.52 (dd, J = 5.1, 1.1 Hz, 1H), 7.44 (dd, J = 3.6, 1.2 Hz, 1H), 7.31‒7.21 (m, 4H), 7.15 (dd, J = 5.1, 3.6 Hz, 1H), 7.08‒6.98 (m, 6H), 2.50 (s, 6H). ^13^C NMR (101 MHz, Chloroform-d) δ(ppm): 146.91, 144.58, 143.69, 132.73, 128.99, 128.33, 127.09, 125.39, 125.31, 123.43, 123.31, 55.47, 15.88.

*Synthesis of Bpin-ThSMeTPA*: ThSMeTPA (0.420 g, 1 mmol) was placed in a sintered, dry screwcap Schlenk-tube under nitrogen atmosphere and dissolved in 5 mL dry THF. The reaction mixture was cooled to ‒78℃ with an acetone/dry ice bath and stirred for 10 min at this temperature. nBuLi (0.1 mL, 1.1 mmol) was added dropwise. After stirring the reaction mixture at ‒78℃ for 1 hour, 2-isopropoxy-4,4,5,5-tetramethyl-1,3,2-dioxaborolane (0.21 g, 1.1 mmol) was added dropwise. After the addition, the mixture was stirred at ‒78℃ for additional 1 hour and then left stirring overnight at room temperature. The reaction was poured in H_2_O and extracted with DCM. The collected organic phases were dried over anhydrous MgSO_4_. After the solvent was evaporated, the residue was purified using column chromatography (silica gel, PE: DCM = 2: 1) to obtain product as Bpin-ThSMeTPA (0.465 g, 85.2%). ^1^H NMR (400 MHz, DMSO-d6) δ(ppm): 7.67‒7.57 (m, 2H), 7.55 (d, J = 3.6 Hz, 1H), 7.51 (d, J = 3.6 Hz, 1H), 7.31‒7.24 (m, 4H), 7.09‒7.02 (m, 4H), 7.02‒6.95 (m, 2H), 2.50 (s, 6H), 1.33 (s, 12H). ^13^C NMR (101 MHz, DMSO-d6) δ(ppm): 144.38, 139.18, 133.13, 128.32, 127.51, 125.67, 124.92, 124.71, 122.79, 84.53, 25.11, 15.82.

*Synthesis of ThSMeTPA-Cz-Cl*: To a solution of Bpin-ThSMeTPA (1.20g, 2.2 mmol) and 3,6-dibromo-9H-carbazole (0.48 g, 1 mmol) in 40 mL of toluene/ethanol/ H_2_O (2/2/1), Pd (PPh_3_)_4_ (0.046 g, 0.02 mmol) and K_2_CO_3_ (0.337 g, 6 mmol) were added in a 250 mL Schlenk flask. The mixture was heated to 85 ℃ for 24 hours under nitrogen. After cooling to RT, the reaction mixture was extracted with DCM and H_2_O. The combined organic layer was dried over anhydrous MgSO_4_. After the solvent was evaporated, the residue was purified using column chromatography (silica gel, PE: DCM = 4: 1) to obtain product as ThSMeTPA-Cz-Cl (1.877 g, 80.1%). ^1^H NMR (400 MHz, DMSO-d6) δ(ppm): 8.67 (d, J = 2.1 Hz, 2H), 7.84 (dd, J = 8.6, 1.8 Hz, 2H), 7.75 (d, J = 8.7 Hz, 2H), 7.68‒7.60 (m, 4H), 7.60 (d, J = 3.7 Hz, 2H), 7.49 (d, J = 3.7 Hz, 2H), 7.31‒7.25 (m, 8H), 7.04 (dd, J = 8.9, 7.2 Hz, 12H), 4.86 (s, 2H), 4.12 (t, J = 5.8 Hz, 2H), 2.54 (p, J = 1.9 Hz, 12H). ^13^C NMR (101 MHz, DMSO-d6) δ(ppm): 146.84, 144.58, 143.64, 141.69, 140.66, 132.80, 128.34, 126.65, 126.07, 125.41, 124.23, 123.38, 55.47, 15.86.

*Synthesis of VCz-ThSMeTPA*: ThSMeTPA-Cz-Cl (1.06 g, 1 mmol) were placed in a 250 mL Schlenk flask together with KOH (0.28 g, 5 mmol), hydroquinone (0.033 g, 0.3 mmol), and dissolved in toluene and isopropanol (IPA), The mixture was refluxed for 12 hours at 85 ℃. After colling to RT, the solvent was removed via vacuum distillation, and extracted with DCM and H_2_O. The combined organic layer was dried over anhydrous MgSO_4_. The crude product was poured into methanol with stirring, producing a gray precipitate. The precipitate was washed thoroughly with water and methanol. And then dried under vacuum at 40 ℃ to obtain product as VCz-ThSMeTPA (0.934 g, 90.8%). ^1^H NMR (400 MHz, DMSO-d6) δ(ppm): 8.31 (s, 2H), 7.75 (d, J = 8.6 Hz, 2H), 7.63 (d, J = 8.6 Hz, 2H), 7.52 (d, J = 8.1 Hz, 4H), 7.34 (d, J = 3.4 Hz, 2H), 7.24 (s, 2H), 7.20 (d, J = 8.1 Hz, 8H), 7.09‒7.03 (m, 12H), 5.57 (d, J = 15.8 Hz, 1H), 5.21 (d, J = 9.3 Hz, 1H), 2.49 (d, J = 2.4 Hz, 11H), 1.26 (s, 1H). ^13^C NMR (101 MHz, DMSO-d6) δ(ppm): 142.74, 139.27, 132.22, 128.57, 126.46, 125.01, 124.29, 123.64, 117.76, 111.30, 102.78, 16.87.

*Synthesis of PVCz-ThSMeTPA*: The monomer VCz-ThSMeTPA (200 mg) and 1 wt % AIBN (2 mg) as initiator were dissolved in 1 mL toluene in a 25-mL Schlenk tube. After degassing through four freeze-evacuate-thaw cycles, the reaction was stirred at 60 ℃ for 72 hours. After cooling, the vessel was opened, and the solution was precipitated into hexane and then extracted on a Soxhlet’s extractor with ethanol for 72 hours to obtain product as PVCz-ThSMeTPA (130 mg). *M*_n_ = 25819, *M*_w_ = 52646, PDI = 2.03.

*Synthesis of Br-OMeTPA*: Br-OMeTPA was synthesized according to the similar procedure used for preparation of Br-SMeTPA. ^1^H NMR (400 MHz, Chloroform-d) δ (ppm): 7.25‒7.19 (m, 2H), 7.07‒6.98 (m, 4H), 6.87‒6.75 (m, 6H), 3.79 (s, 6H). ^13^C NMR (101 MHz, Chloroform-d) δ(ppm): 154.16, 148.03, 140.66, 131.86, 126.67, 122.07, 114.87, 112.44, 55.59.

*Synthesis of ThOMeTPA*: ThOMeTPA was synthesized according to the similar procedure used for preparation of ThSMeTPA. ^1^H NMR (400 MHz, Chloroform-d) δ(ppm): 7.44‒7.38 (m, 2H), 7.20‒7.16 (m, 2H), 7.09‒7.04 (m, 5H), 6.96‒6.89 (m, 2H), 6.85‒6.82 (m, 4H), 3.80 (s, 6H). ^13^C NMR (101 MHz, Chloroform-d) δ(ppm): 156.02, 148.28, 144.73, 140.81, 128.00, 126.69, 126.67, 123.60, 121.80, 120.70, 114.81, 55.59

*Synthesis of Bpin-ThOMeTPA*: Bpin-ThOMeTPA was synthesized according to the similar procedure used for preparation of Bpin-ThSMeTPA. ^1^H NMR (400 MHz, Chloroform-d) δ(ppm): 7.56 (d, J = 3.6 Hz, 1H), 7.48‒7.37 (m, 2H), 7.25 (d, J = 3.6 Hz, 1H), 7.08 (d, J = 8.9 Hz, 4H), 6.93‒6.88 (m, 2H), 6.86‒6.83 (m, 4H), 3.80 (s, 6H). ^13^C NMR (101 MHz, Chloroform-d) δ(ppm): 156.17, 151.78, 148.70, 140.62, 138.34, 126.92, 126.88, 126.28, 123.17, 120.24, 114.84, 84.12, 55.98, 24.86.

*Synthesis of ThOMeTPA-Cz-Cl*: ThOMeTPA-Cz-Cl was synthesized according to the similar procedure used for preparation of ThSMeTPA-Cz-Cl. ^1^H NMR (400 MHz, DMSO-d6) δ(ppm): 8.61 (d, J = 1.9 Hz, 2H), 7.79 (dd, J = 8.6, 1.8 Hz, 2H), 7.70 (d, J = 8.7 Hz, 2H), 7.52 (dd, J = 9.6, 3.0 Hz, 6H), 7.37 (d, J = 3.8 Hz, 2H), 7.10‒7.02 (m, 8H), 6.98‒6.89 (m, 8H), 6.80 (d, J = 8.6 Hz, 4H), 4.82 (d, J = 6.3 Hz, 2H), 4.08 (t, J = 5.8 Hz, 2H), 3.75 (s, 12H). ^13^C NMR (101 MHz, DMSO-d6) δ(ppm): 156.43, 148.35, 142.96, 142.18, 140.58, 127.38, 126.44, 126.17, 126.09, 123.74, 119.92, 115.55, 55.78, 55.47.

*Synthesis of VCz-ThOMeTPA*: VCz-ThOMeTPA was synthesized according to the similar procedure used for preparation of VCz-ThSMeTPA. ^1^H NMR (400 MHz, Chloroform-d) δ(ppm): 8.31 (dd, J = 1.9, 0.6 Hz, 2H), 7.75 (dd, J = 8.6, 1.9 Hz, 2H), 7.66‒7.59 (m, 2H), 7.49‒7.45 (m, 4H), 7.34‒7.26 (m, 3H), 7.20 (d, J = 3.8 Hz, 2H), 7.12‒7.07 (m, 8H), 6.97‒6.93 (m, 4H), 6.87‒6.83 (m, 8H), 5.56 (dd, J = 15.9, 1.1 Hz, 1H), 5.20 (dd, J = 9.2, 1.1 Hz, 1H), 3.81 (s, 12H), 1.26 (s, 1H). ^13^C NMR (101 MHz, Chloroform-d) δ(ppm): 156.03, 148.20, 143.25, 142.94, 140.80, 139.18, 127.81, 126.73, 126.28, 124.68, 124.47, 123.43, 122.77, 120.72, 117.32, 114.81, 111.05, 55.61.

*Synthesis of PVCz-ThOMeTPA*: PVCz-ThOMeTPA was synthesized according to the similar procedure used for preparation of PVCz-ThSMeTPA. *M*_n_ = 19304, *M*_w_ = 63494, PDI = 3.28.

*Materials Characterization*: All the solvents were treated according to the standard procedures. ^1^H and ^13^C NMR in CDCl_3_ were recorded at 400 MHz using a Bruker 400 MHz spectrometermrelative to tetramethylsilane (TMS) as internal standard. The Shimadzu DSC-60A and Shimadzu DTG-60H were used to conduct TG analysis and differential scanning calorimetry of materials at a heating rate of 10 °C min^‒1^ under N_2_. The absorption and PL emission spectra of the materials were recorded by Shimadzu UV-1780 spectrophotometer and Hitachi F-4600 spectrophotometer, respectively. Cyclic voltammetry studies were performed using a CHI660E system in a typical three-electrode cell with a glassy carbon working electrode, a platinum wire counter electrode, and a silver/silver chloride (Ag/AgCl) reference electrode. All electrochemical experiments were performed at room temperature in a nitrogen atmosphere in an electrolyte solution of 0.1M tetrabutylammonium hexafluorophosphatate (Bu_4_NPF_6_) at a scanning rate of 0.1 V s^‒1^. The electrochemical potential was calibrated using ferrocene/ferrocene (Fc/Fc+) couple as an external standard. The ionization energy measurement system IPS-4 (Nanjing Sunny Tech Co, LTD) was used to analyze the Photoelectron Yield Spectroscopy (PYS) under N_2_. Steady-state PL spectra were measured using a QE65 Pro spectrometer and a 445 nm CW laser as the excitation source. Lifetime, time-resolved emission spectra were obtained on an Edinburgh FLSP920 fluorescence spectrophotometer equipped with a xenon arc lamp (Xe900), a nanosecond hydrogen flash lamp (nF920) and a microsecond flash lamp (μF900). XRD data were obtained using an X-ray diffractometer (Rigaku Smart Laboratory XRD). XPS experiments were conducted using the Thermo Scientific Escalab XI+spectrometer and Magcis Ar+/Arn+GCIS gun from the United States. The scanning electron microscope images of perovskite films were characterized by high resolution field emission top view scanning electron microscope (JSM-7800F). Atomic force microscopy (AFM) images were acquired in non-contact mode (Park XE7). Contact angles were analyzed using a microscopic contact angle meter (DSA100, Kruss).

*Devices characterization*: Perovskite solar cells were measured and characterized under a solar simulator (Class AAA, SS-F5-3A, Enlitech), simulated AM1.5G 100 mW cm^−2^ illumination, and recorded the current density-voltage (*J‒V*) curve with a Keithley 2450 source meter. The stabilized power output at the MPP of the solar cells, the light intensity uses the Xingang standard silicon cell 91150 for calibration. The IPCE measurement was carried out in DC mode using a Kessley 2400 source instrument and a SOFN71SW752 monochromator equipped with a 500W xenon lamp. The Hamamatsu S1337-1010BQ silicon diode was calibrated at the National Institute of Metrology of China. EIS measurement was recorded on CHI760E electrochemical workstation (CH Instruments Ins, USA).

*Hole-only device measurements*: The hole-transporting properties of HTMs were evaluated by using the space charge limitation of the current method under dark condition. The hole-only device for hole mobility measurements consists of ITO/PEDOT: PSS/HTM/MoO_3_/Au. Pretched ITO substrates were treated with plasma for 15 minutes. Spin 40 nm PEDOT: PSS layer on ITO substrate, then anneal at 150 °C for 20 minutes The HTMs were dissolved in anhydrous chlorobenzene (30 mg ml^‒1^ for HTMs), spin-coated onto the PEDOT: PSS layer at 3000 rpm. The thicknesses of the films are certified by P-7 Stylus Profiler. A bilayer cathode structure of MoO_3_ (5 nm)/Au (100 nm) was thermally evaporated on top of the HTM layer. *J‒V* characteristics of the devices have been measured with Keithley 2450 Source-Measure unit, interfaced with a computer. The structure of the hole-only device is ITO/PEDOT: PSS with and without polymer/perovskite /PTAA/Ag. The hole defects in the device are calculated, and the density of hole-trap states is determined by the TFL region.

**Cost analysis**

**Table S1.** Synthesis cost of PVCz-ThSMeTPA

| Chemical name | Weight of reagent(g) | Price  ($/g) | Cost  ($) | Cost  ($) |
| --- | --- | --- | --- | --- |
| 4-Iodothioanisole | 1.170 | 3.978 | 4.654 | 12.414 |
| 4-Bromoaniline | 0.860 | 0.700 | 0.602 |  |
| 1,10-Phenanthroline | 0.018 | 0.549 | 0.010 |  |
| CuCl | 0.001 | 0.024 | 0.001 |  |
| Toluene | 129.900 | 0.005 | 0.650 |  |
| 2-Thiopheneboronic acid | 0.130 | 0.701 | 0.091 |  |
| Pd (PPh_3_)_4_ | 0.092 | 17.836 | 1.641 |  |
| K_2_CO_3_ | 0.617 | 0.005 | 0.003 |  |
| THF | 4.450 | 0.015 | 0.066 |  |
| n-Butyllithium | 0.068 | 0.201 | 0.013 |  |
| 2-isopropoxy-4,4,5,5-tetramethyl-1,3,2-dioxaborolane | 0.210 | 0.782 | 0.164 |  |
| 3,6-Diiodo-9H-carbazole | 0.480 | 0.450 | 0.216 |  |
| Ethanol | 86.823 | 0.003 | 0.260 |  |
| KOH | 1.400 | 0.005 | 0.007 |  |
| Hydroquinone | 0.033 | 0.031 | 0.001 |  |
| IPA | 39.275 | 0.019 | 0.746 |  |
| Methanol | 39.550 | 0.009 | 0.356 |  |
| AIBN | 0.002 | 0.035 | 0.001 |  |
| Petroleum ether  (recyclable) | 1100 | 0.0014 | 1.540 |  |
| DCM  (recyclable) | 500 | 0.0014 | 0.700 |  |
| N-hexane | 32.950 | 0.021 | 0.692 |  |

**Table S2.** Synthesis cost of PVCz-ThOMeTPA

| Chemical name | Weight of reagent(g) | Price  ($/g) | Cost  ($) | Cost  ($) |
| --- | --- | --- | --- | --- |
| 4-Iodoanisole | 1.170 | 0.676 | 0.791 | 8.551 |
| 4-Bromoaniline | 0.860 | 0.700 | 0.602 |  |
| 1,10-Phenanthroline | 0.018 | 0.549 | 0.010 |  |
| CuCl | 0.001 | 0.024 | 0.001 |  |
| Toluene | 129.9 | 0.005 | 0.650 |  |
| 2-Thiopheneboronic acid | 0.130 | 0.701 | 0.091 |  |
| Pd (PPh_3_)_4_ | 0.092 | 17.836 | 1.641 |  |
| K_2_CO_3_ | 0.617 | 0.005 | 0.003 |  |
| THF | 4.450 | 0.015 | 0.066 |  |
| n-Butyllithium | 0.068 | 0.201 | 0.013 |  |
| 2-isopropoxy-4,4,5,5-tetramethyl-1,3,2-dioxaborolane | 0.210 | 0.782 | 0.164 |  |
| 3,6-Diiodo-9H-carbazole | 0.480 | 0.450 | 0.216 |  |
| Ethanol | 86.823 | 0.003 | 0.260 |  |
| KOH | 1.400 | 0.005 | 0.007 |  |
| Hydroquinone | 0.033 | 0.031 | 0.001 |  |
| IPA | 39.275 | 0.019 | 0.746 |  |
| Methanol | 39.550 | 0.009 | 0.356 |  |
| AIBN | 0.002 | 0.035 | 0.001 |  |
| Petroleum ether  (recyclable) | 1100 | 0.0014 | 1.540 |  |
| DCM  (recyclable) | 500 | 0.0014 | 0.700 |  |
| N-hexane | 32.950 | 0.021 | 0.692 |  |


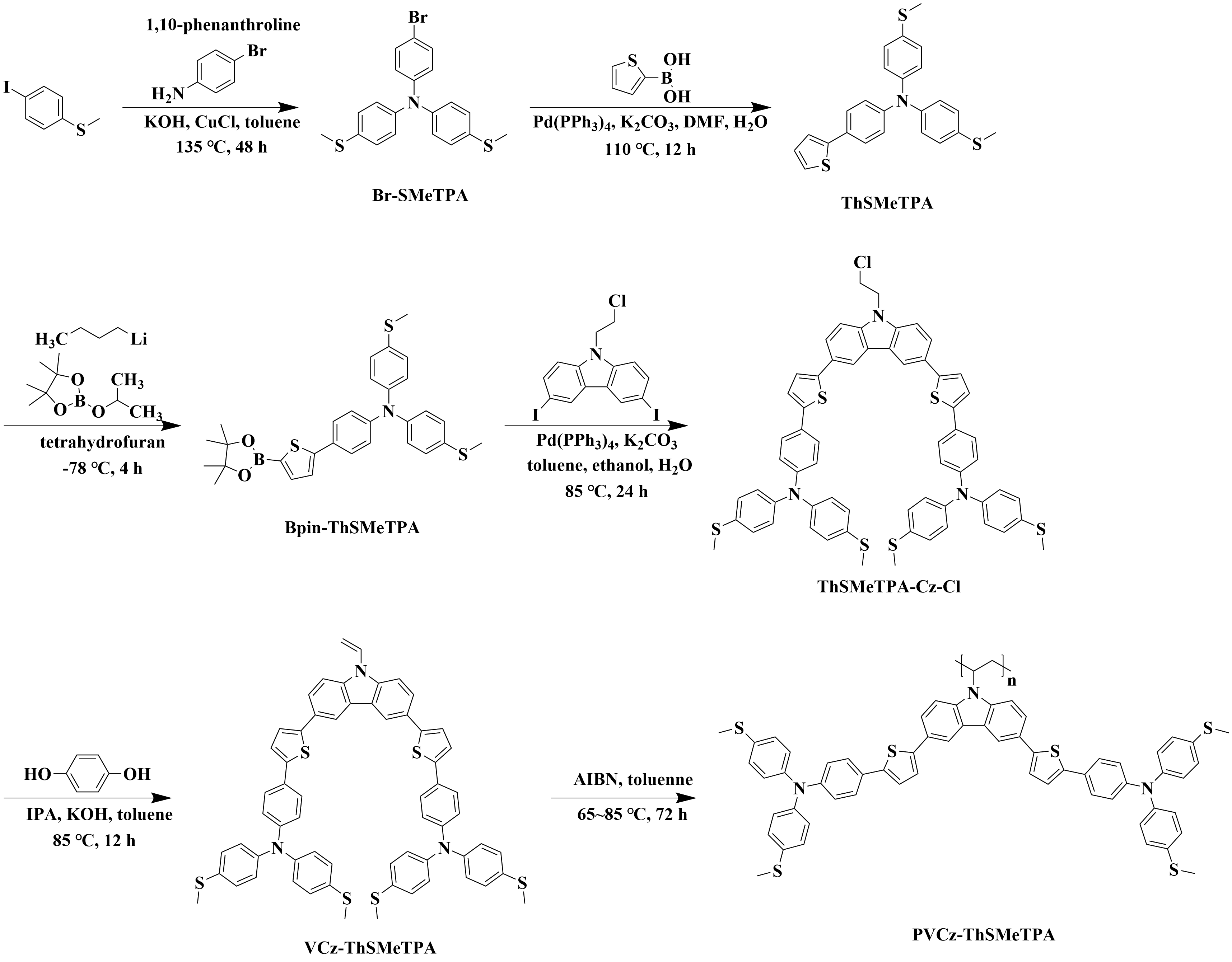


Figure S1. Synthetic routes of PVCz-ThSMeTPA


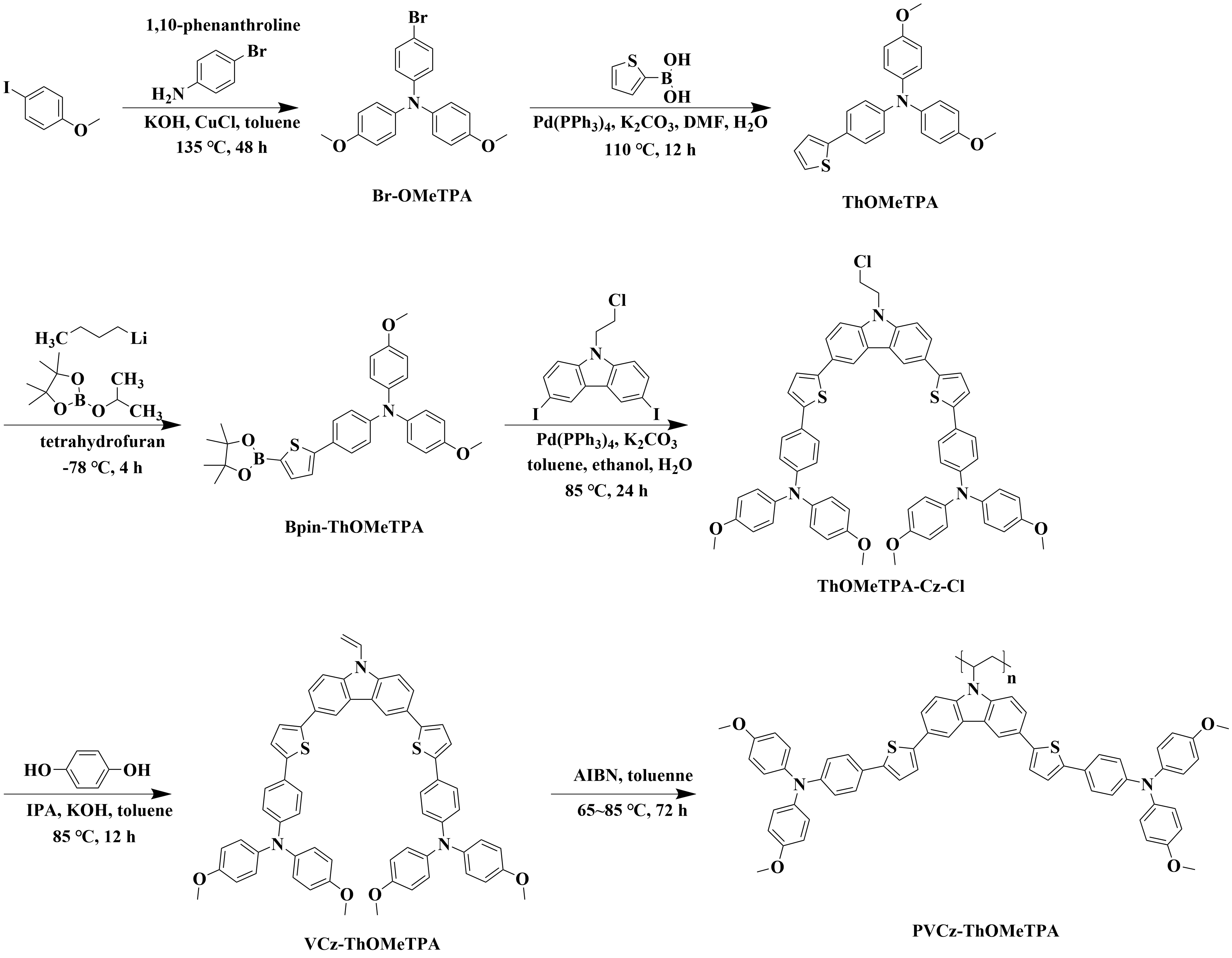


Figure S2. Synthetic routes of PVCz-ThOMeTPA


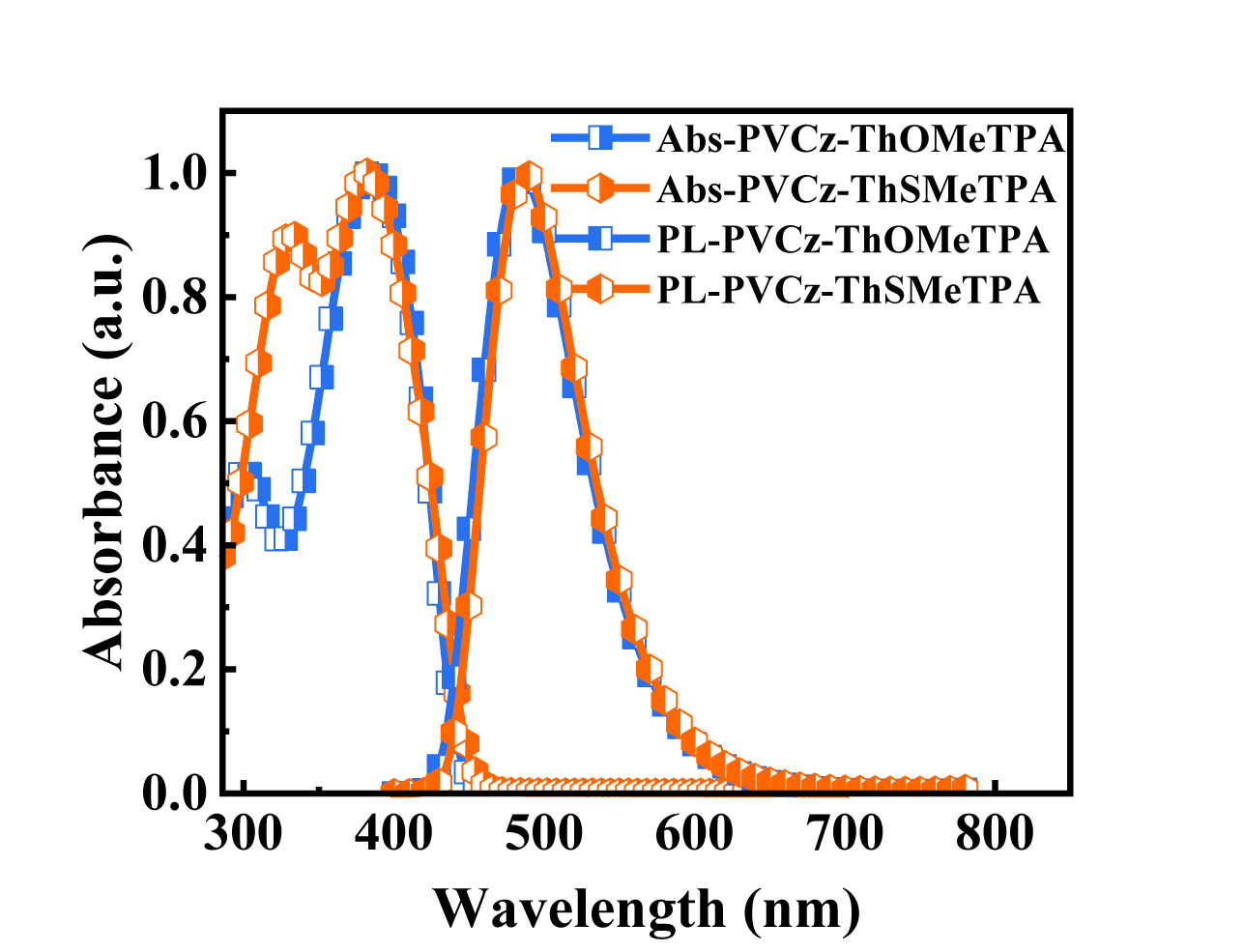


**Figure S3**. Normalized UV-Vis absorption-PL emission spectra of PVCz-ThSMeTPA and PVCz-ThOMeTPA solutions.


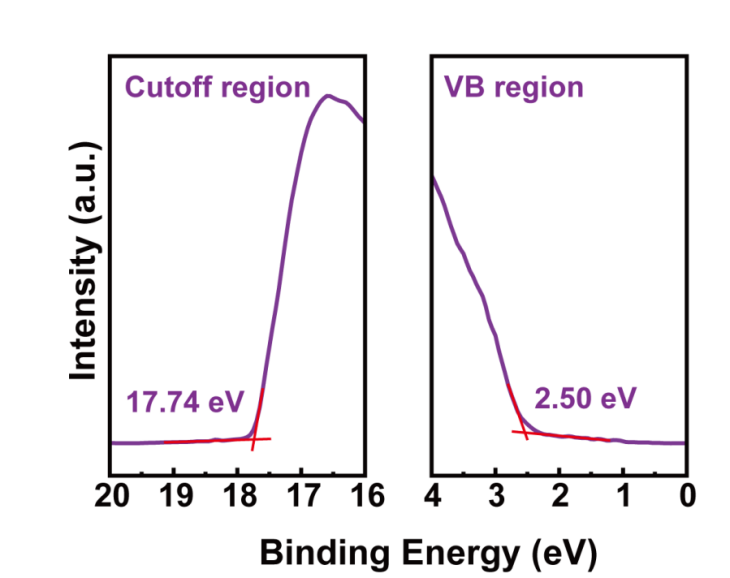


**Figure S4**. UPS spectra of the quasi-2D perovskite film.


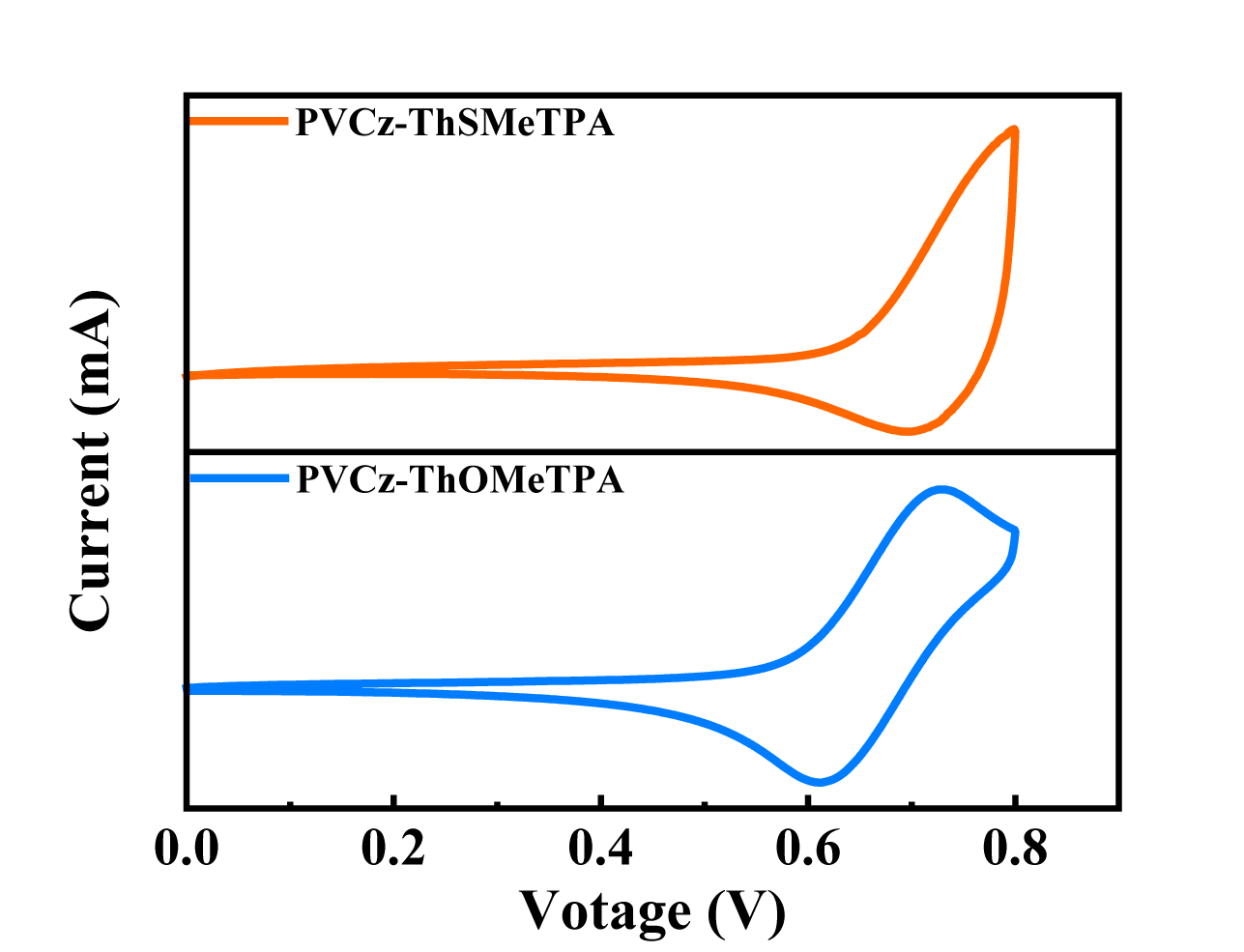


**Figure S5**. Cyclic voltammetry curves of PVCz-ThSMeTPA and PVCz-ThOMeTPA films


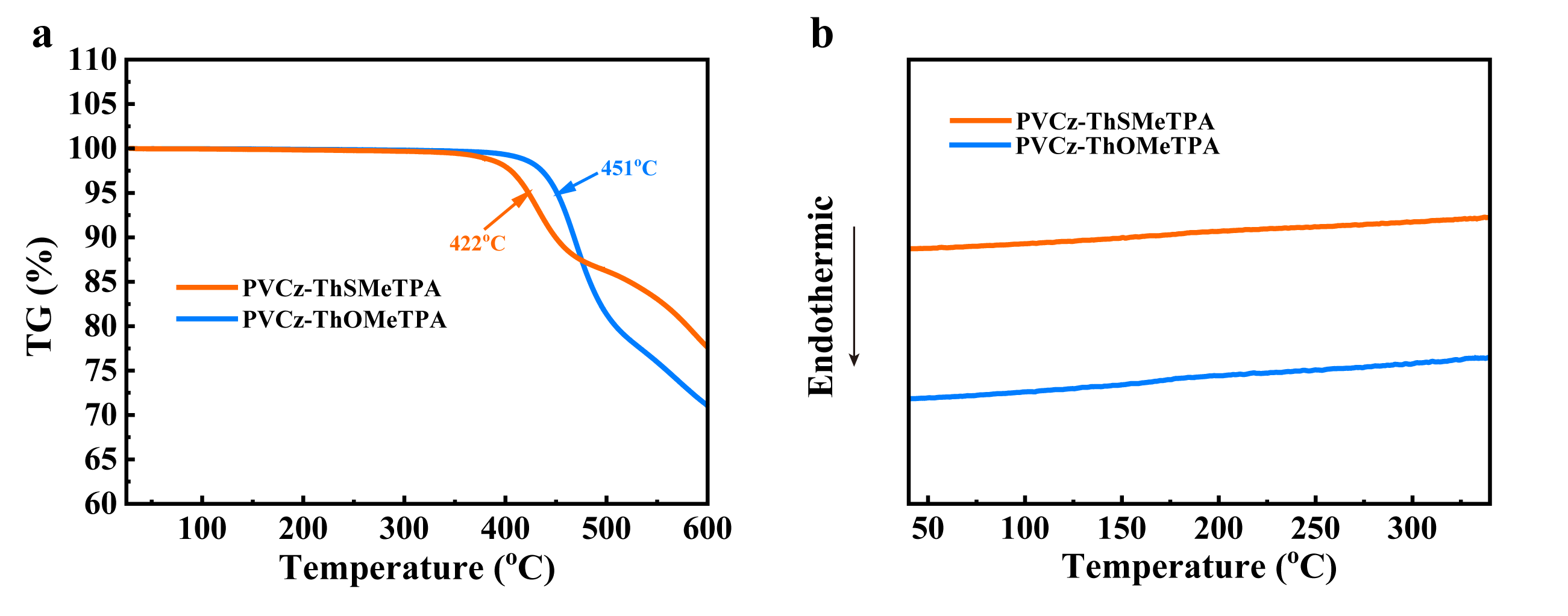


**Figure S6.** TGA and DSC curves of PVCz-ThSMeTPA and PVCz-ThOMeTPA


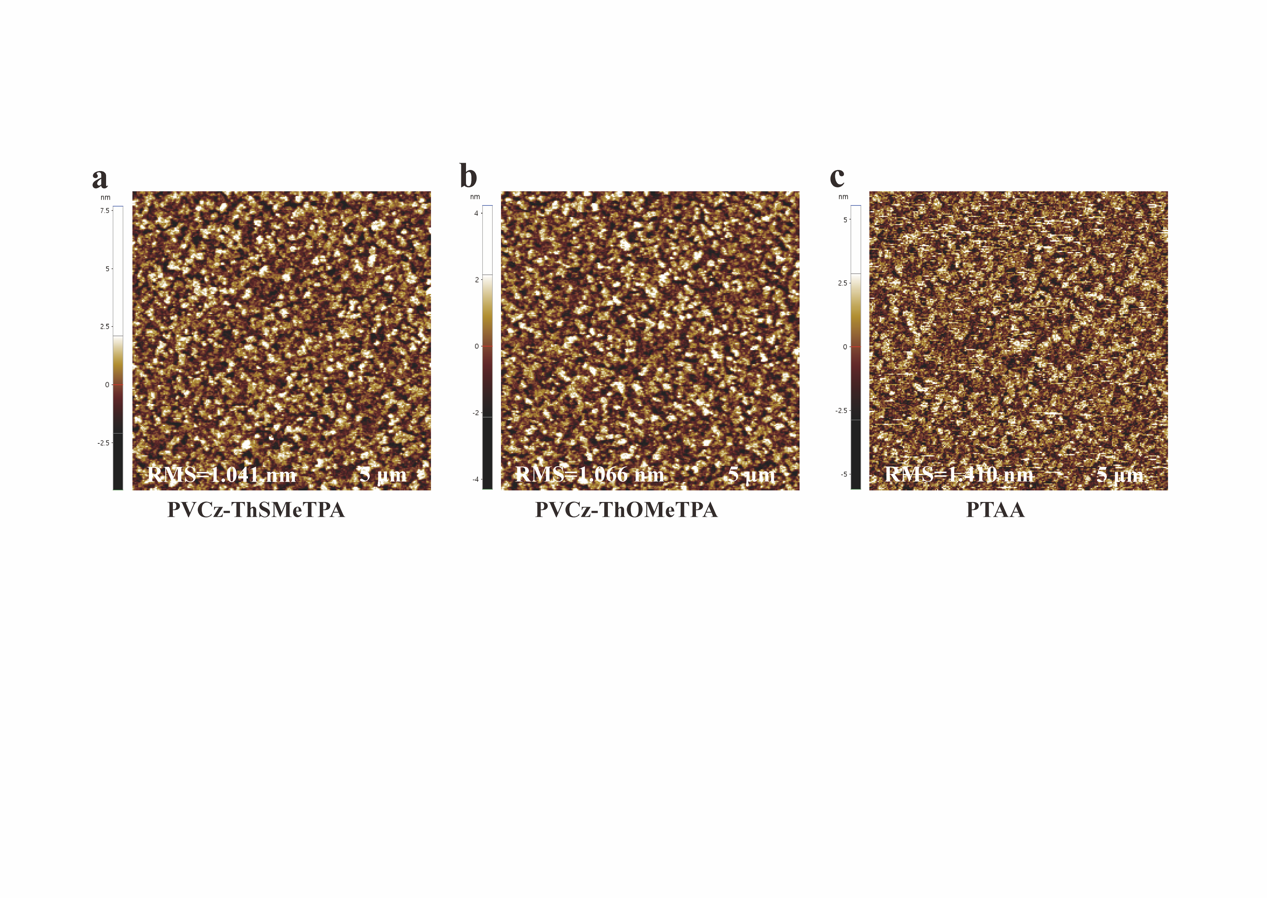


**Figure S7**. AFM images of PVCz-ThSMeTPA, PVCz-ThOMeTPA, and PTAA


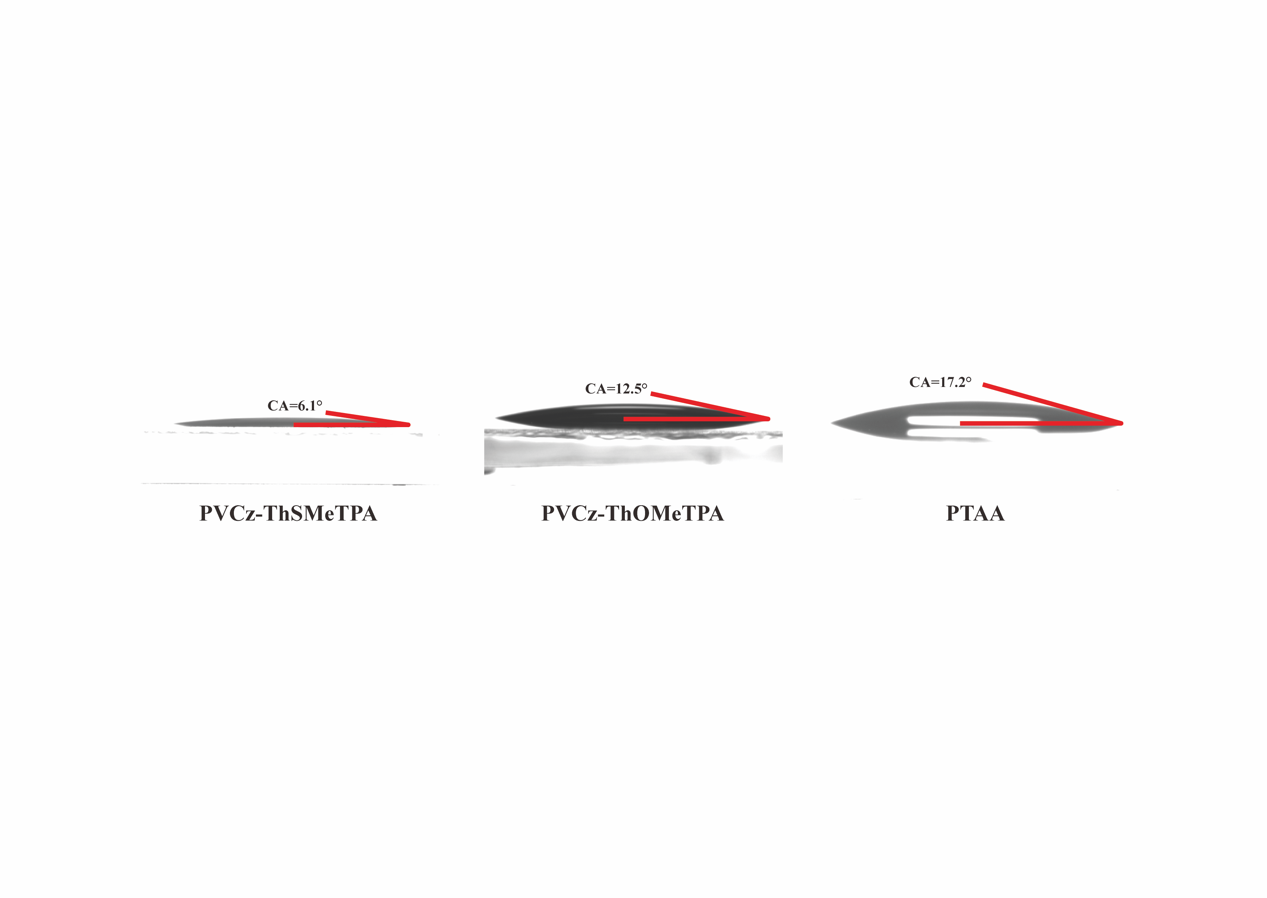


**Figure S8**. Water contact angle of PVCz-ThSMeTPA, PVCz-ThOMeTPA, and PTAA.


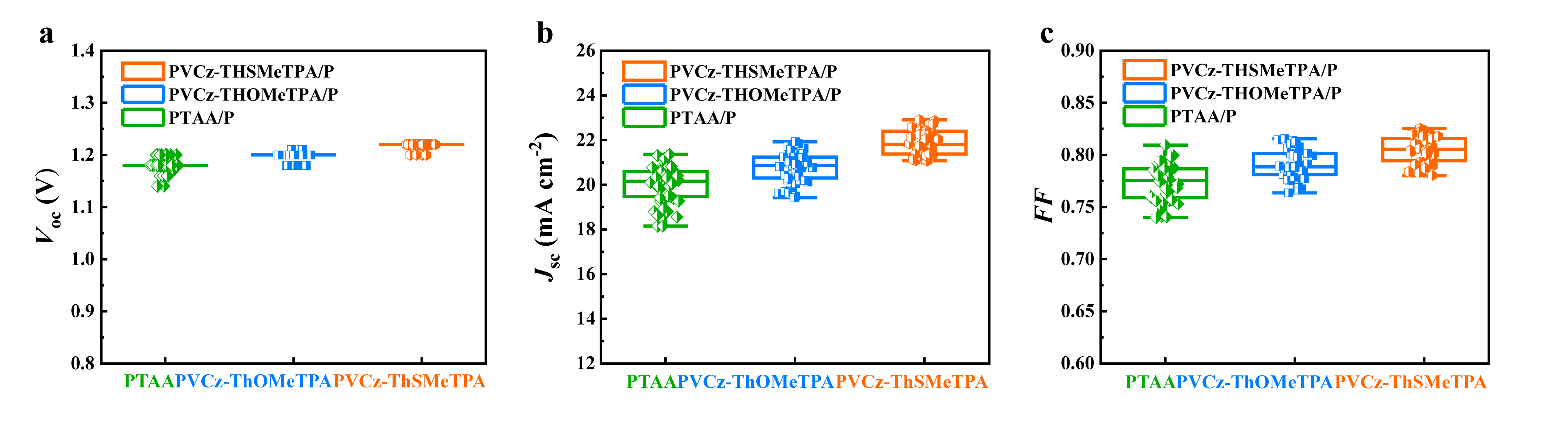


**Figure S9**. *V*_oc_, *J*_sc_ and *FF* statistics of perovskite solar cells based on PVCz-ThSMeTPA, PVCz-ThOMeTPA and PTAA.


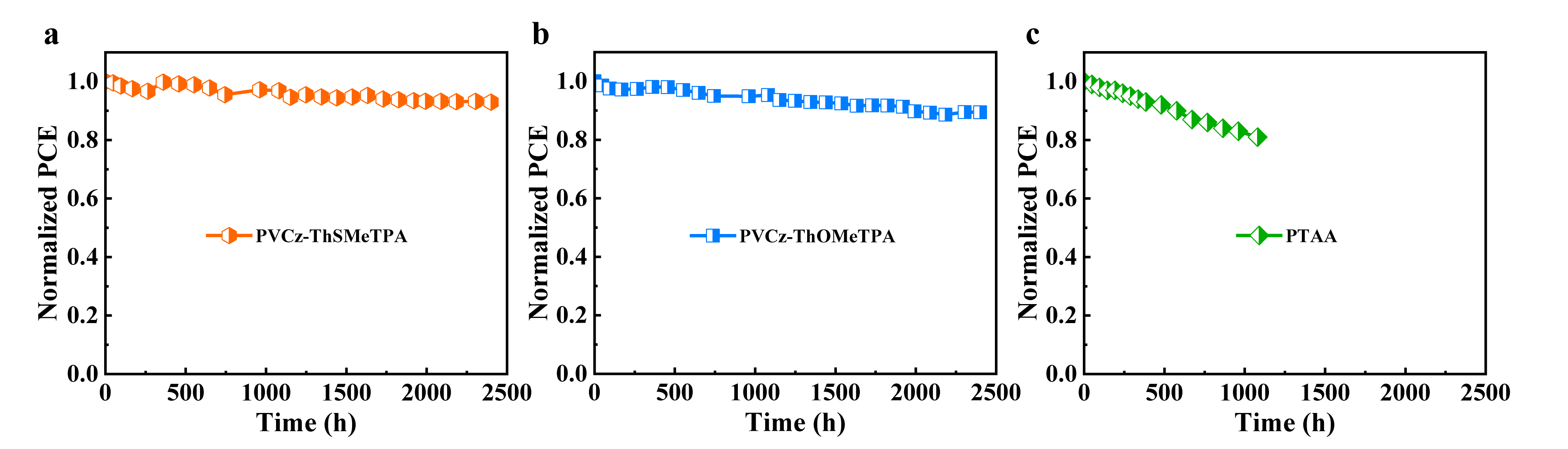


**Figure S10.** Stability tests of a) PVCz-ThSMeTPA, b) PVCz-ThsMeTPA and c) PTAA based inverted quasi-2D PSCs kept in inert atmosphere for long-term stability.

Table S3. Fitting parameters of the TRPL spectra for the pristine and HTMs modified perovskite films based on the results of Figure 6b.

| HTMs | 𝜏1(ns) | 𝜏2(ns) |
| --- | --- | --- |
| PVCz-ThSMeTPA | 4.35 | 46.06 |
| PVCz-ThOMeTPA | 6.95 | 74.43 |
| PTAA | 14.34 | 86.99 |
| perovskite | 16.99 | 113.30 |


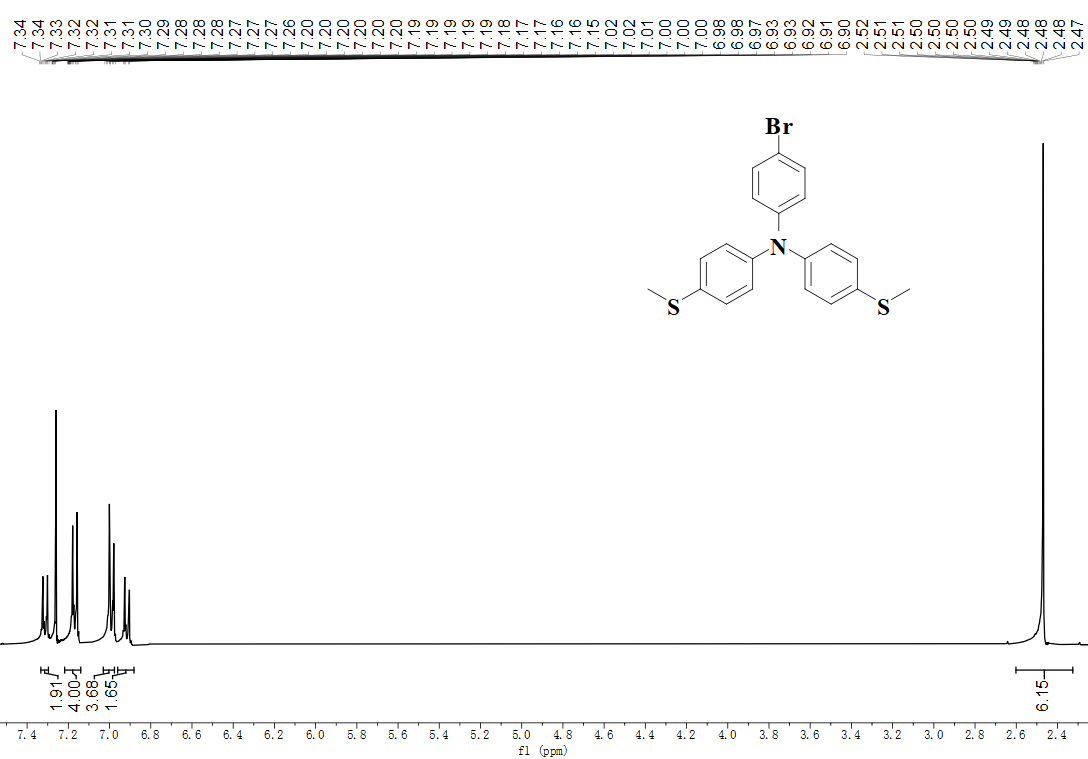


**Figure S11**. ^1^H NMR spectrum of Br-SMeTPA.


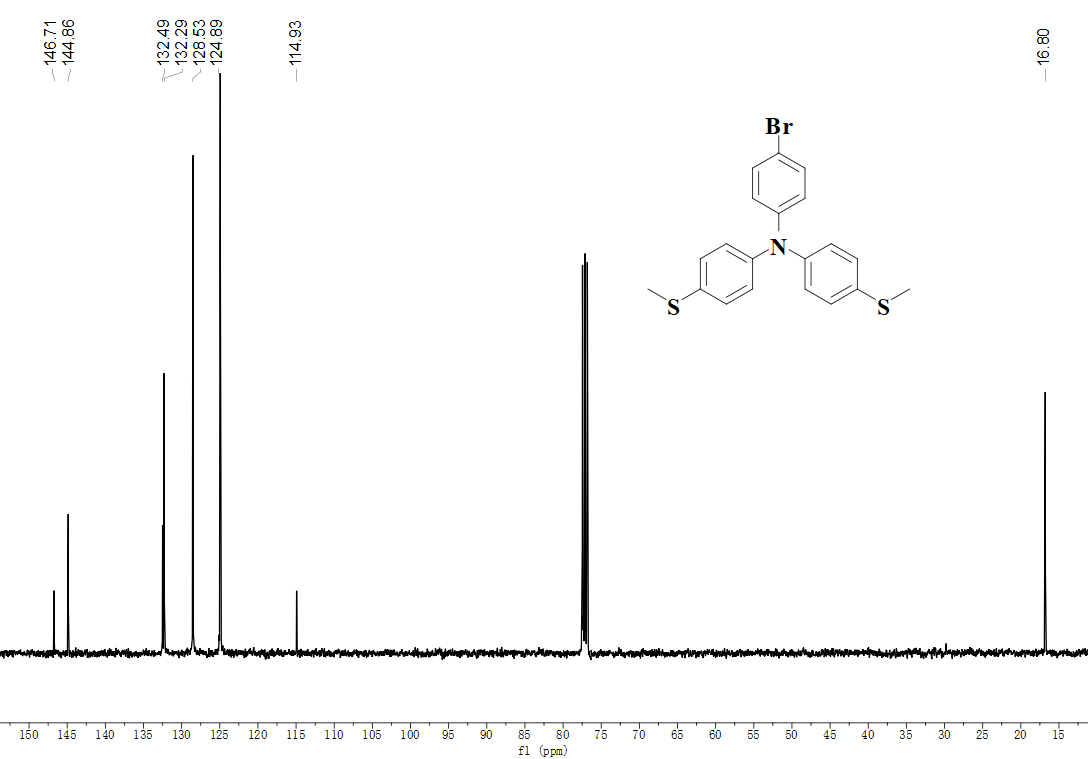


**Figure S12**. ^13^C NMR spectrum of Br-SMeTPA.


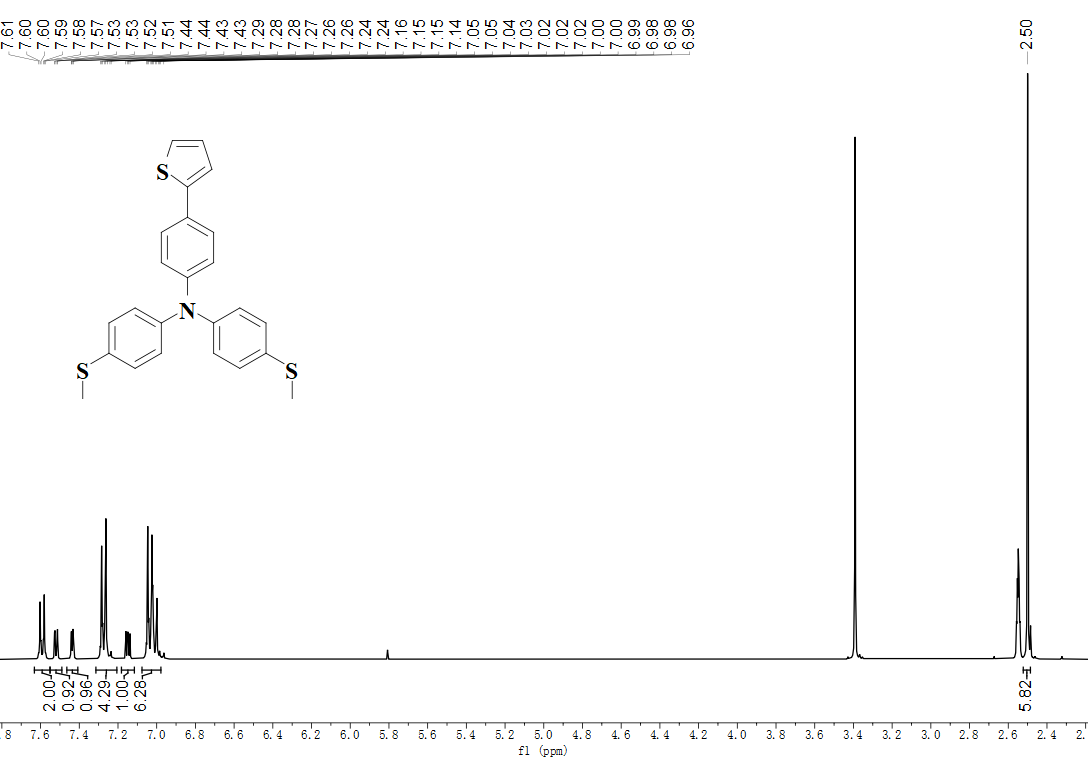


**Figure S13**. ^1^H NMR spectrum of ThSMeTPA.


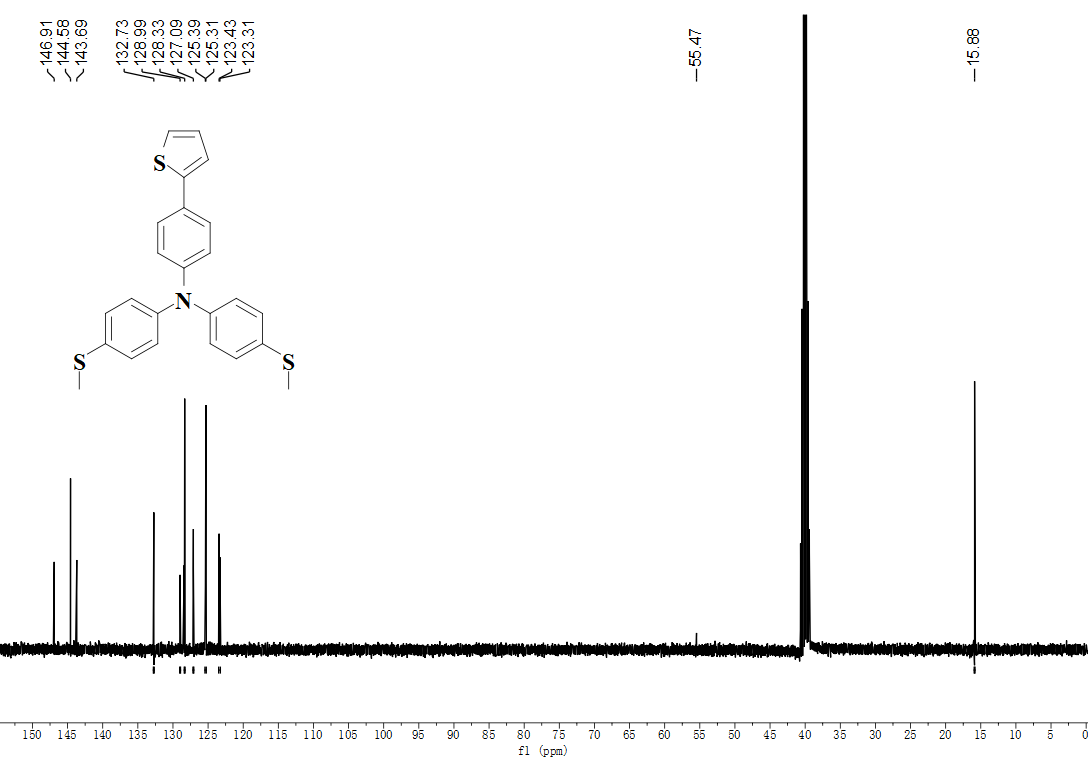


**Figure S14**. ^13^C NMR spectrum of ThSMeTPA.


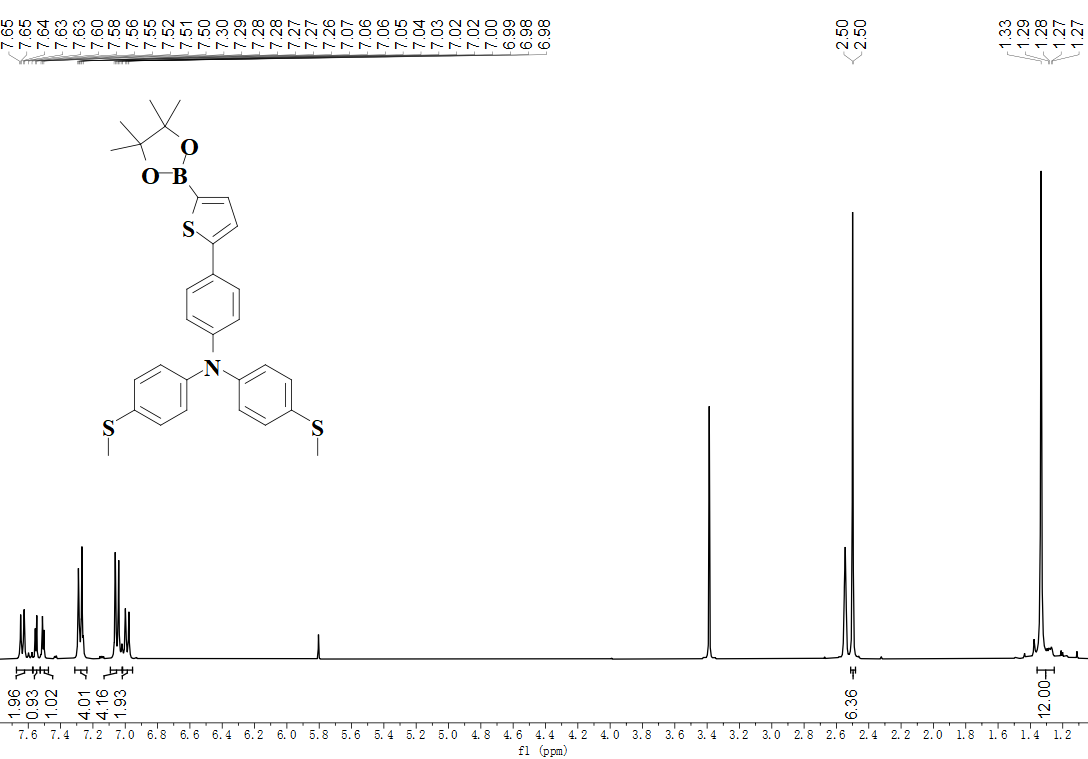


**Figure S15**. ^1^H NMR spectrum of Bpin-ThSMeTPA.


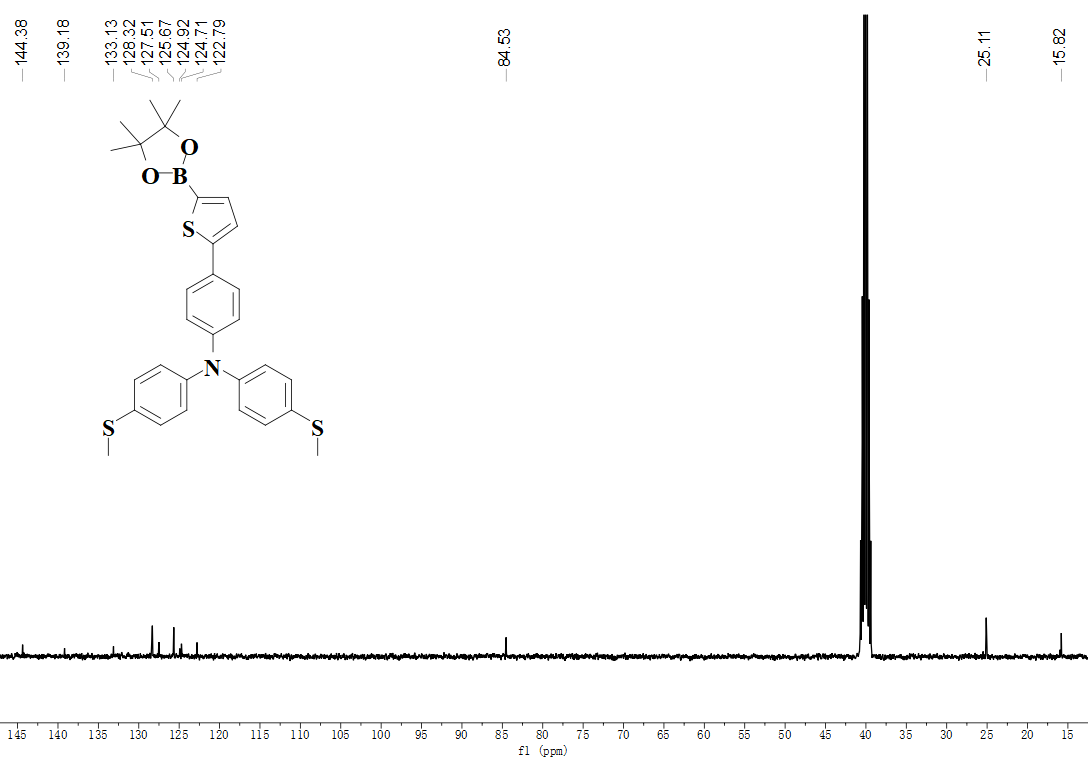


**Figure S16**. ^13^C NMR spectrum of Bpin-ThSMeTPA.


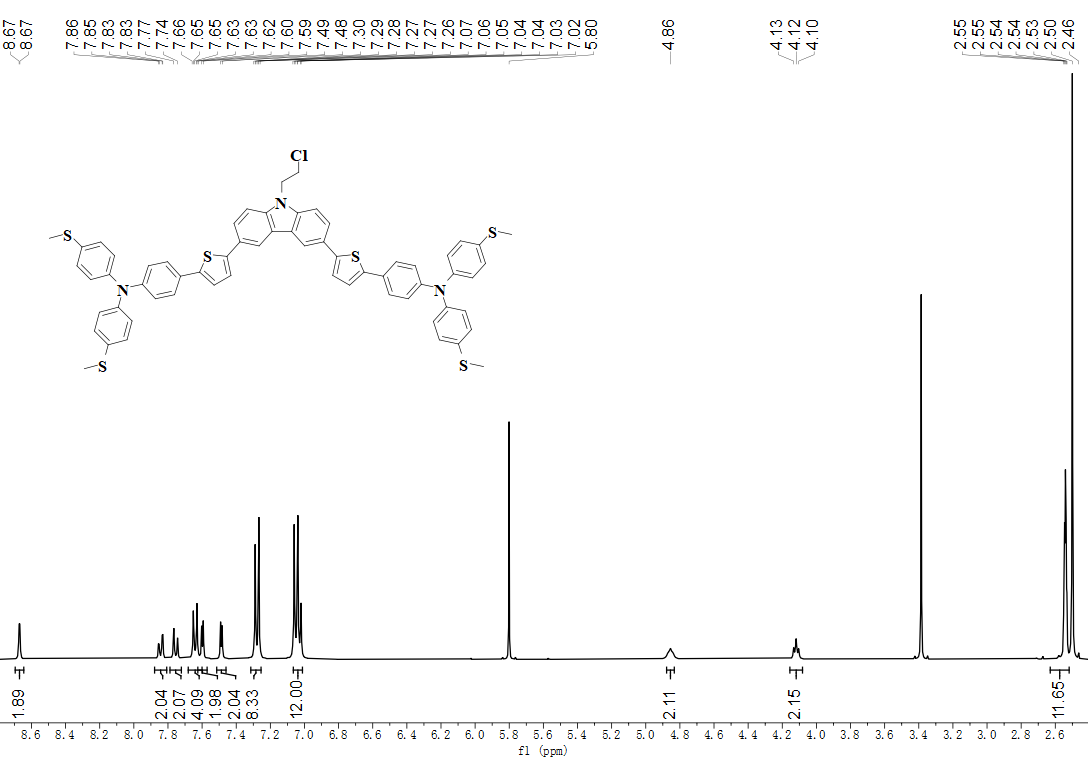


**Figure S17**. ^1^H NMR spectrum of ThSMeTPA-Cz-Cl.


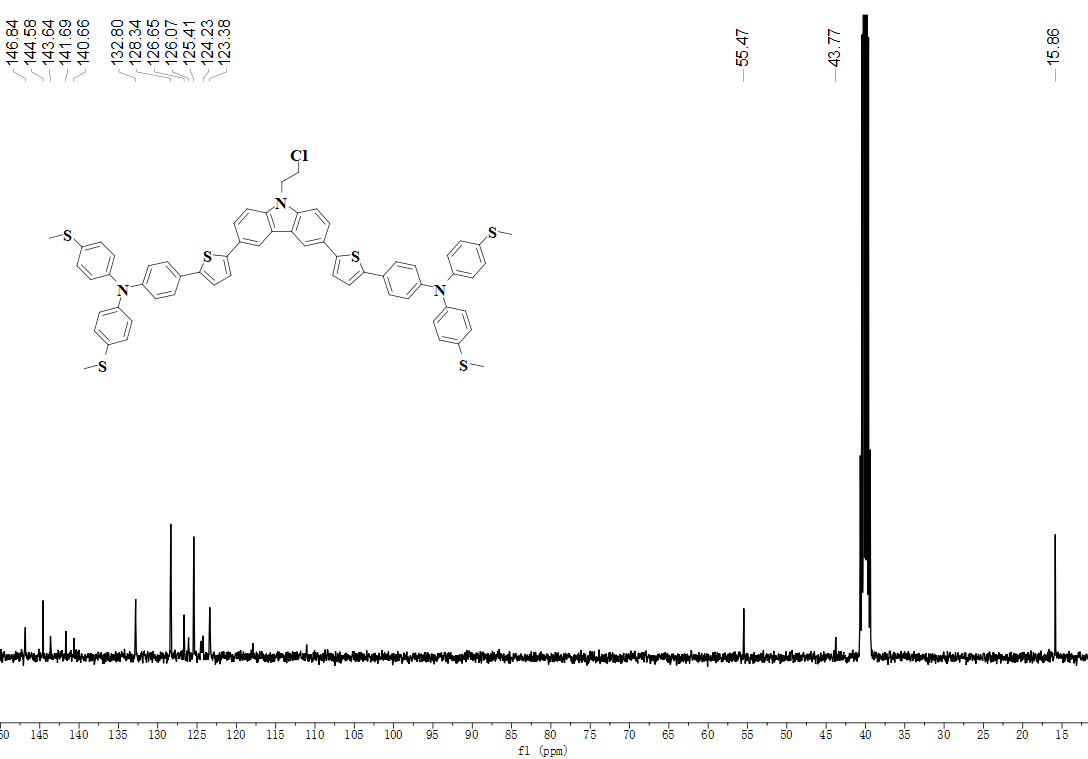


**Figure S18**. ^13^C NMR spectrum of ThSMeTPA-Cz-Cl.


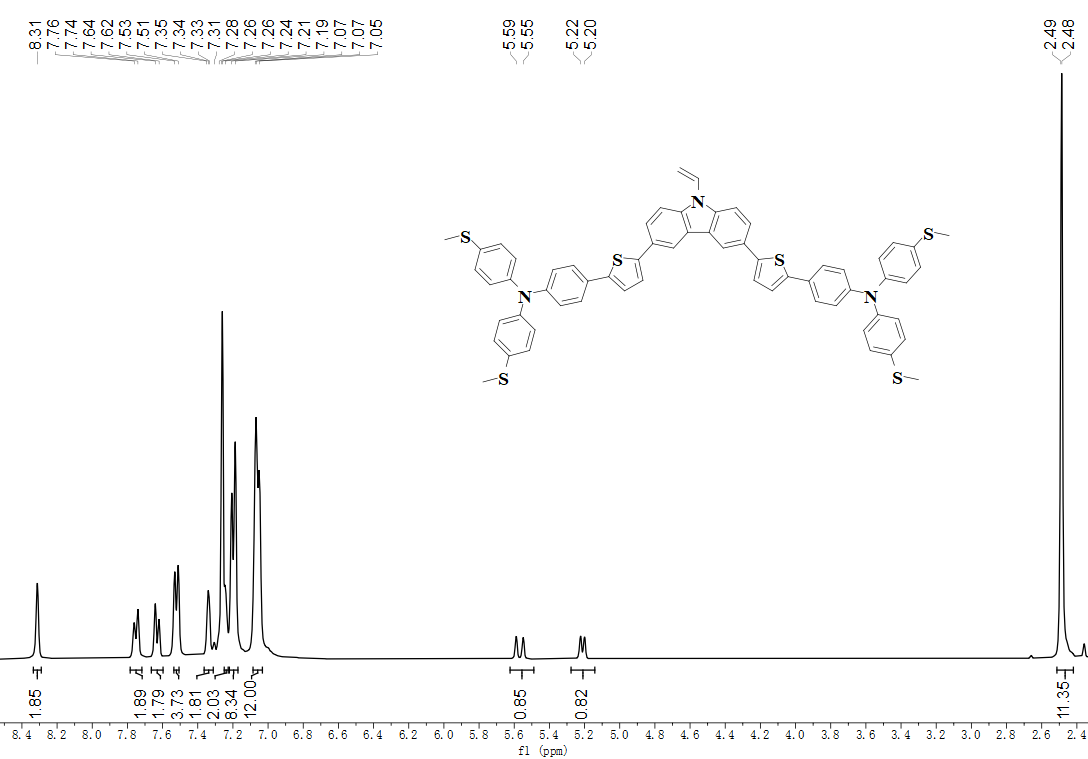


**Figure S19**. ^1^H NMR spectrum of VCz-ThSMeTPA.


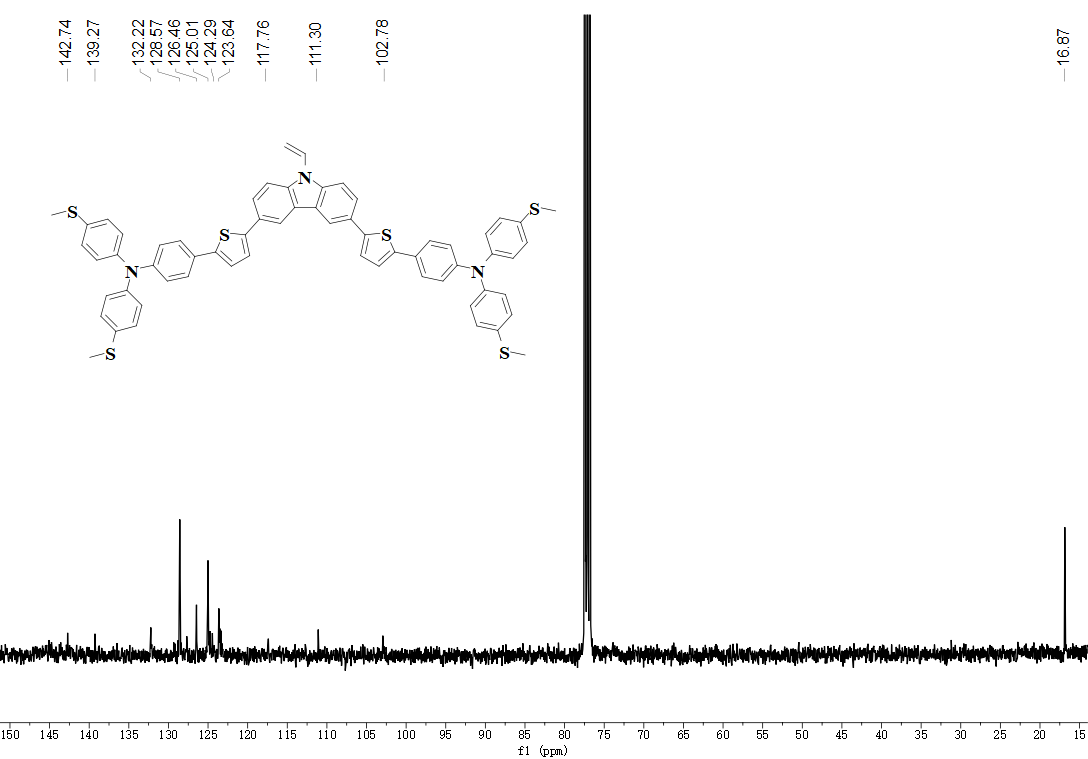


**Figure S20**. ^13^C NMR spectrum of VCz-ThSMeTPA.


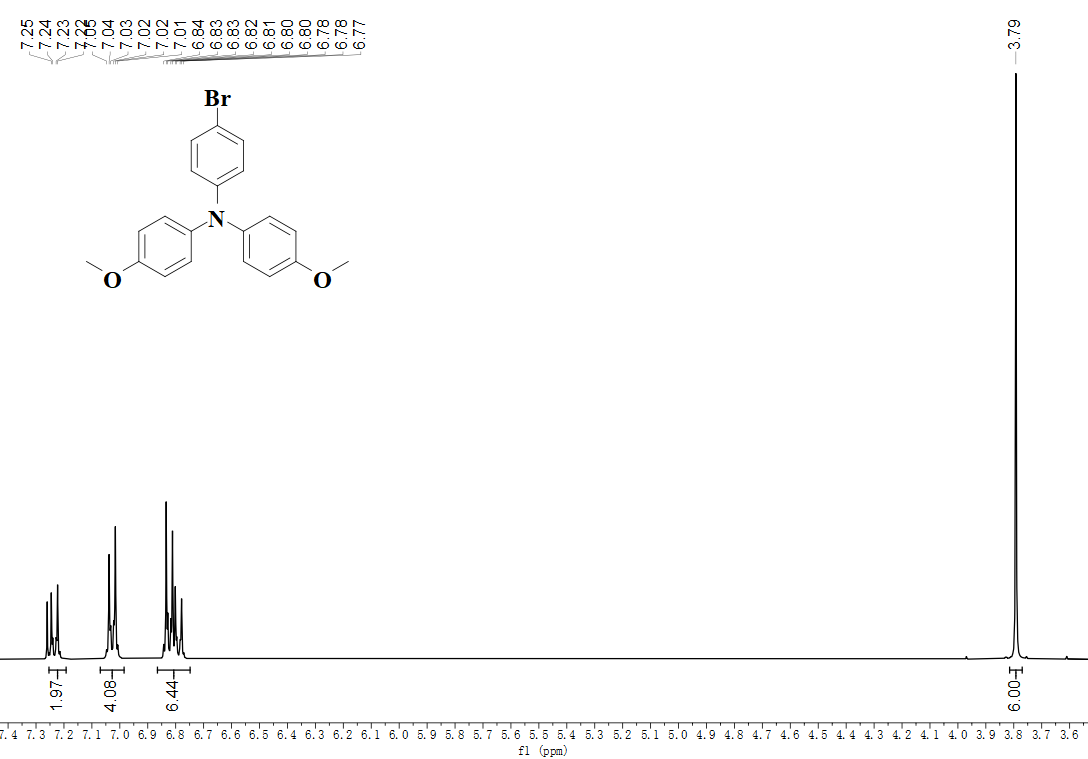


**Figure S21**. ^1^H NMR spectrum of Br-OMeTPA.


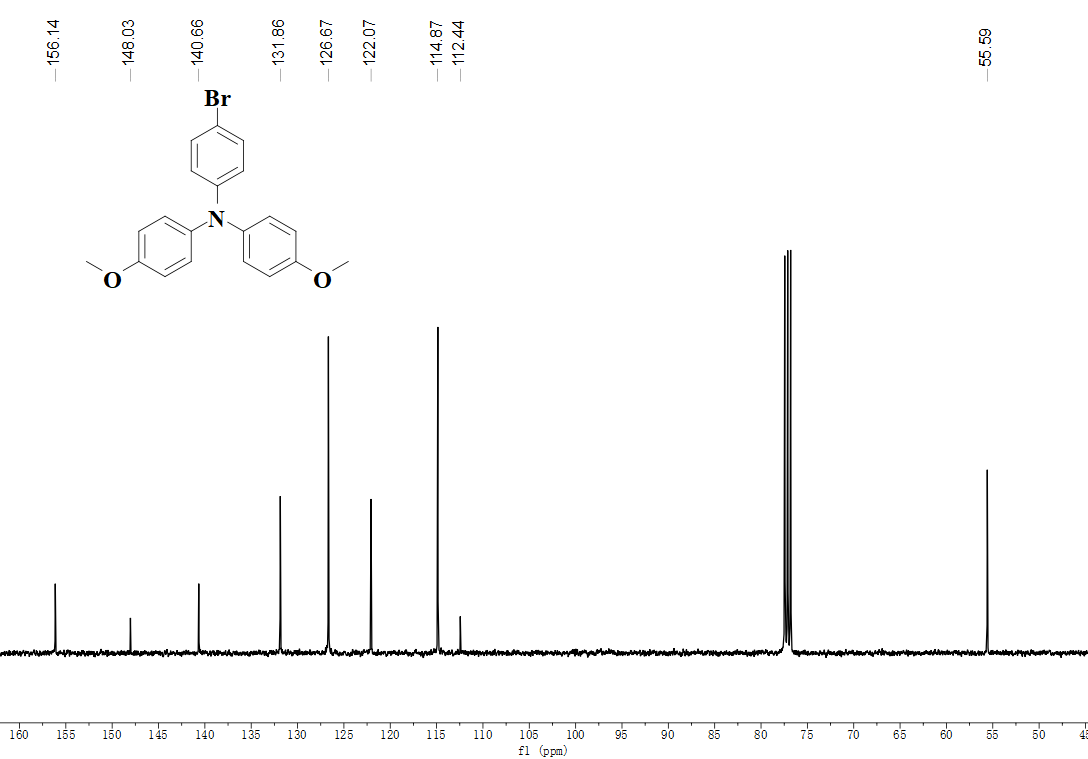


**Figure S22**. ^13^C NMR spectrum of Br-OMeTPA.


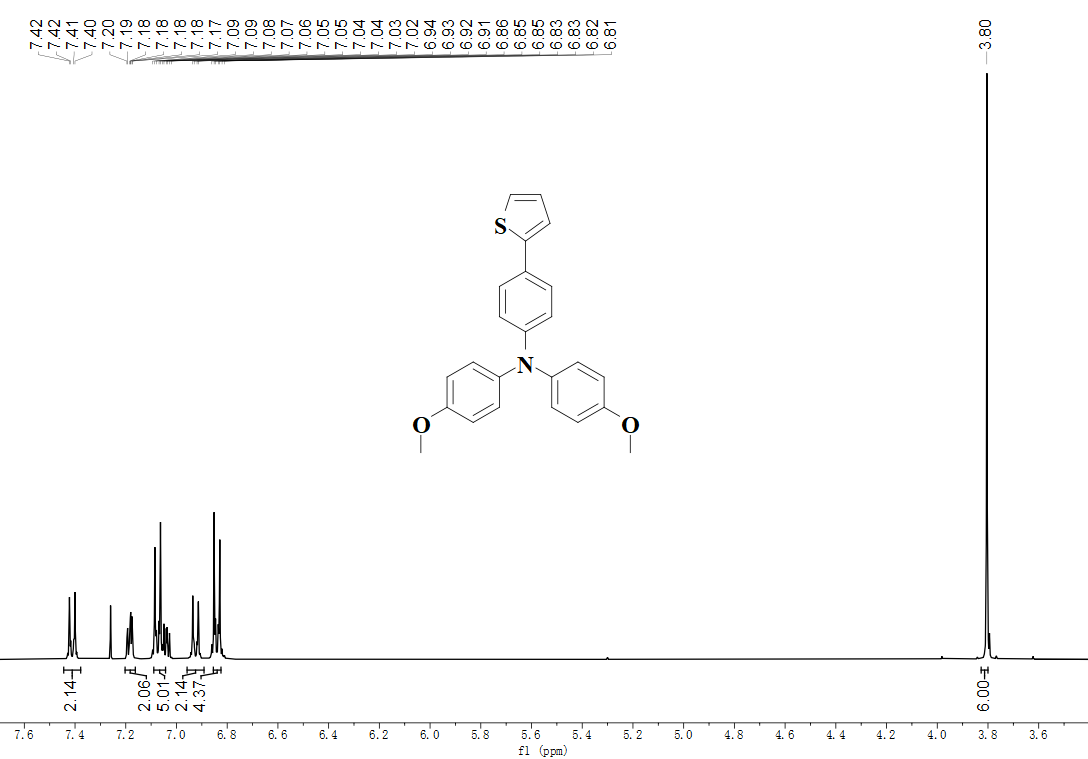


**Figure S23**. ^1^H NMR spectrum of ThOMeTPA.


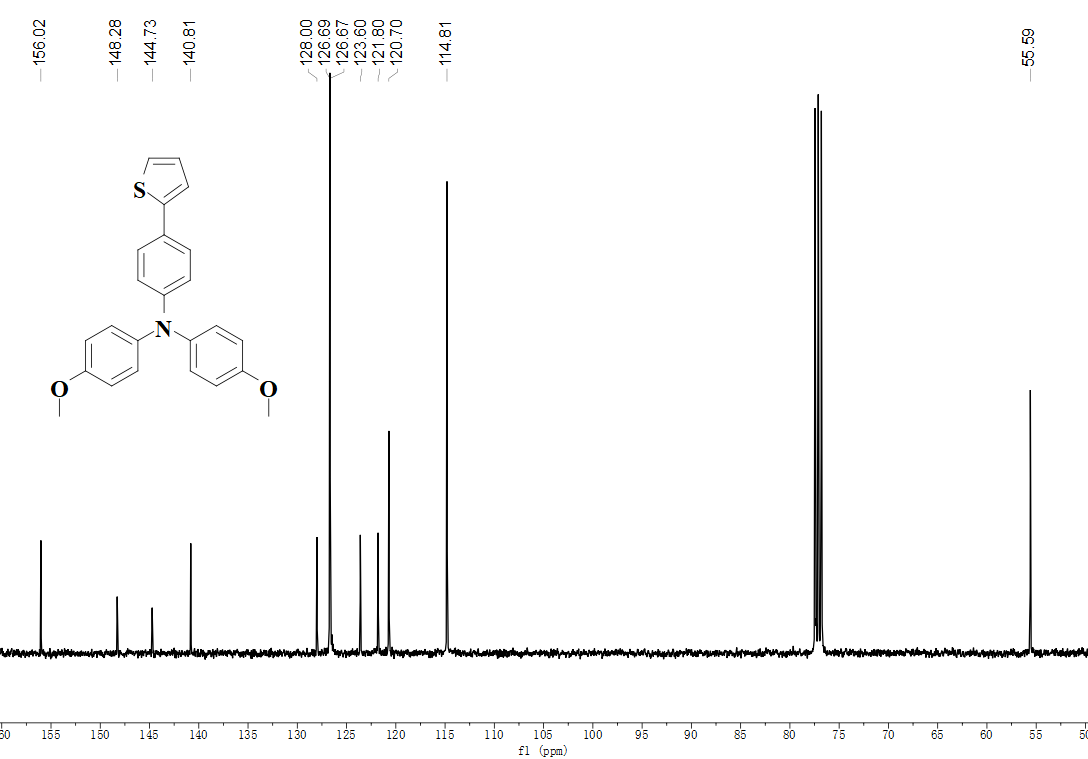


**Figure S24**. ^13^C NMR spectrum of ThOMeTPA.


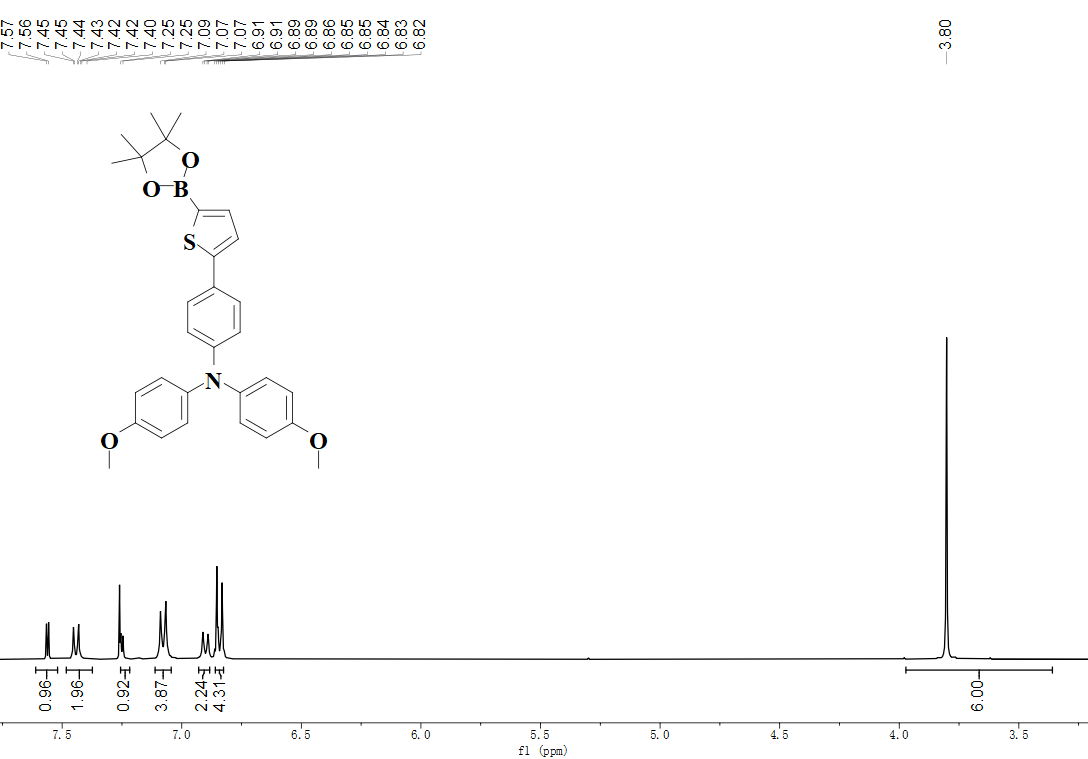


**Figure S25**. ^1^H NMR spectrum of Bpin-ThOMeTPA.


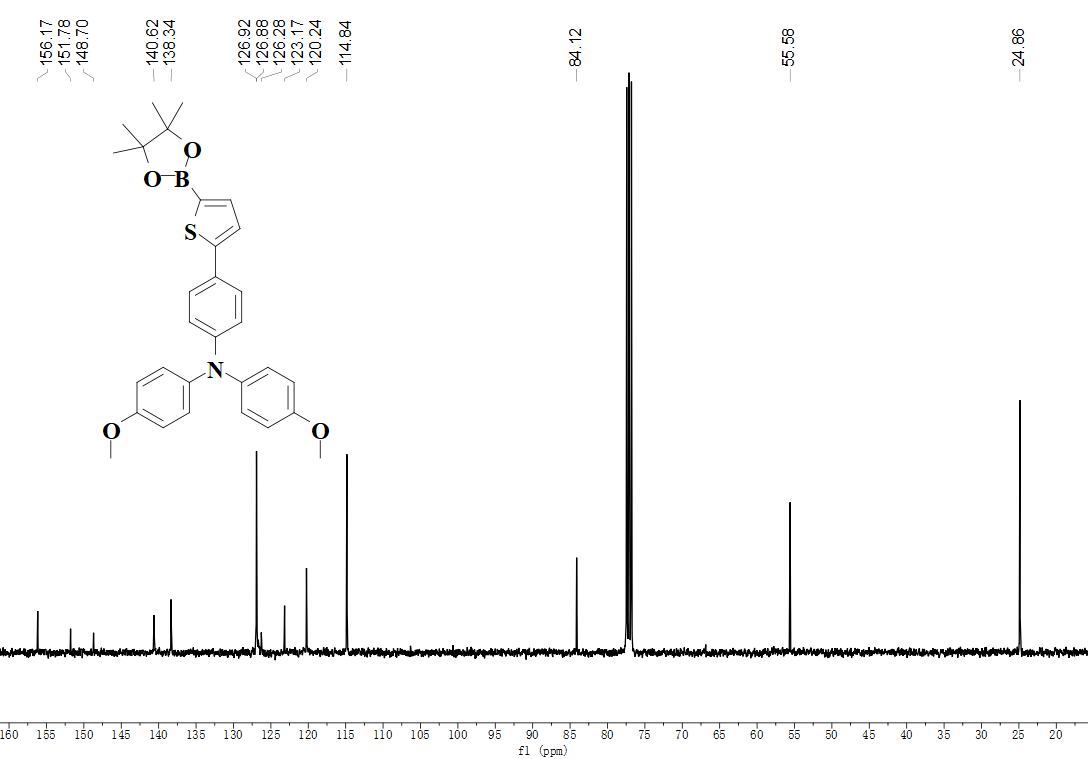


**Figure S26**. ^13^C NMR spectrum of Bpin-ThOMeTPA.


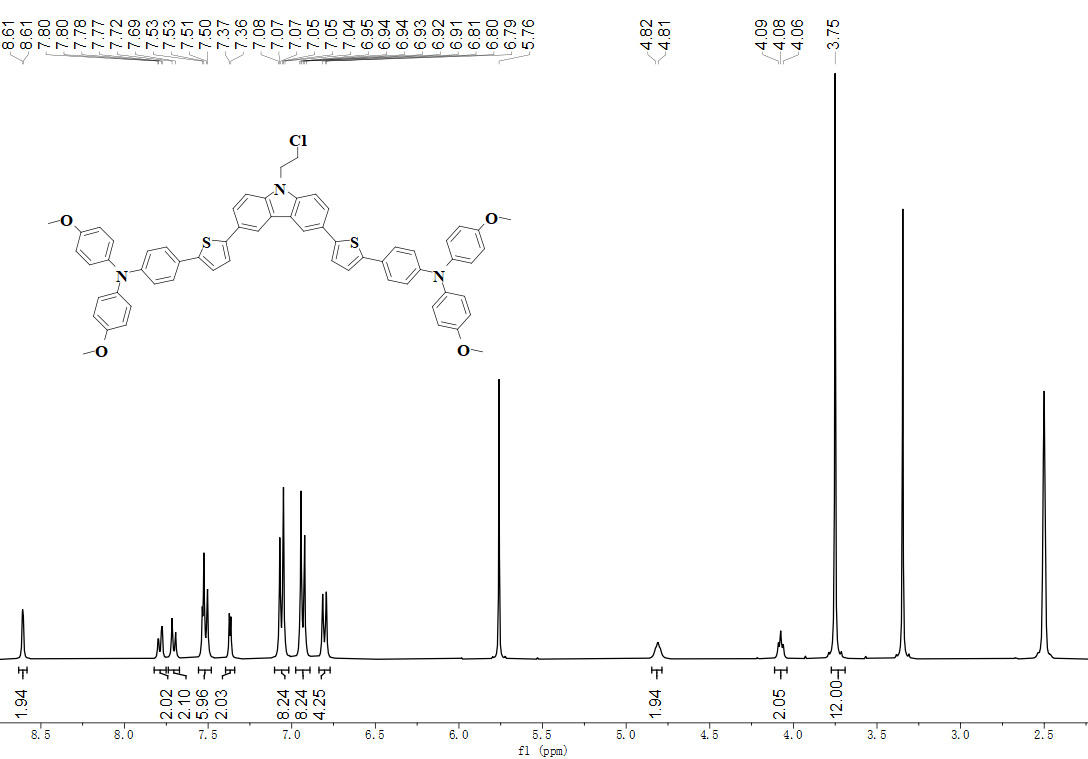


**Figure S27**. ^1^H NMR spectrum of ThOMeTPA-Cz-Cl.


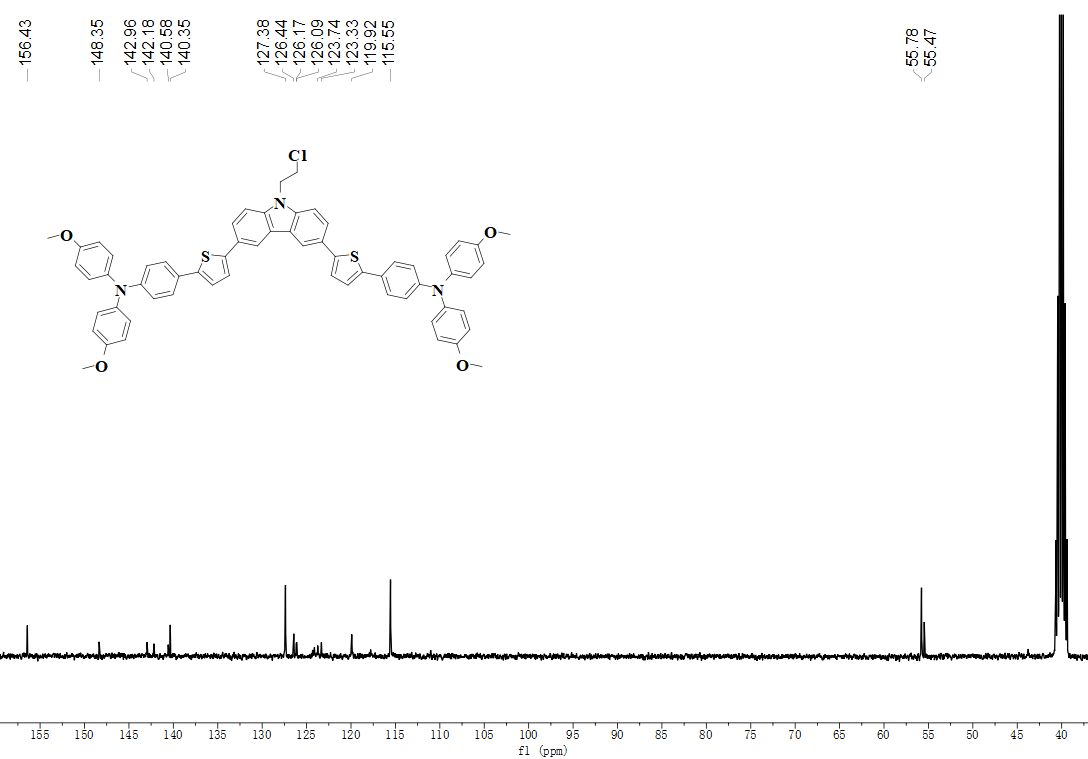


**Figure S28**. ^13^C NMR spectrum of ThOMeTPA-Cz-Cl.


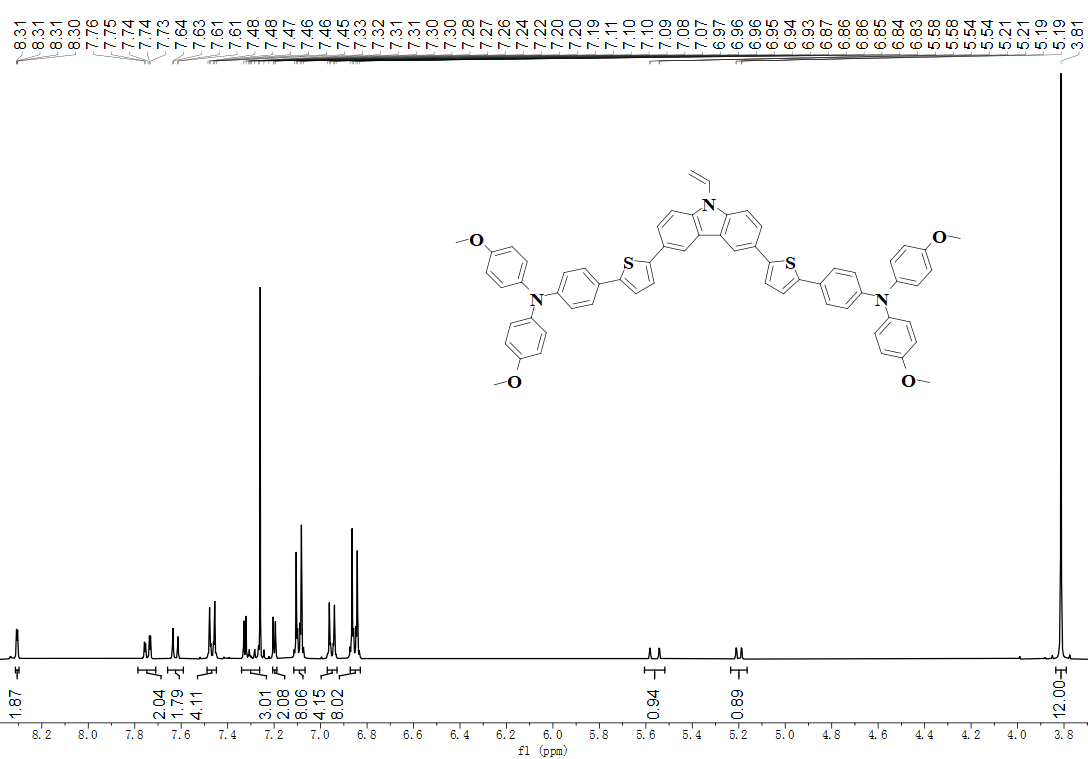


**Figure S29**. ^1^H NMR spectrum of VCz-ThOMeTPA.


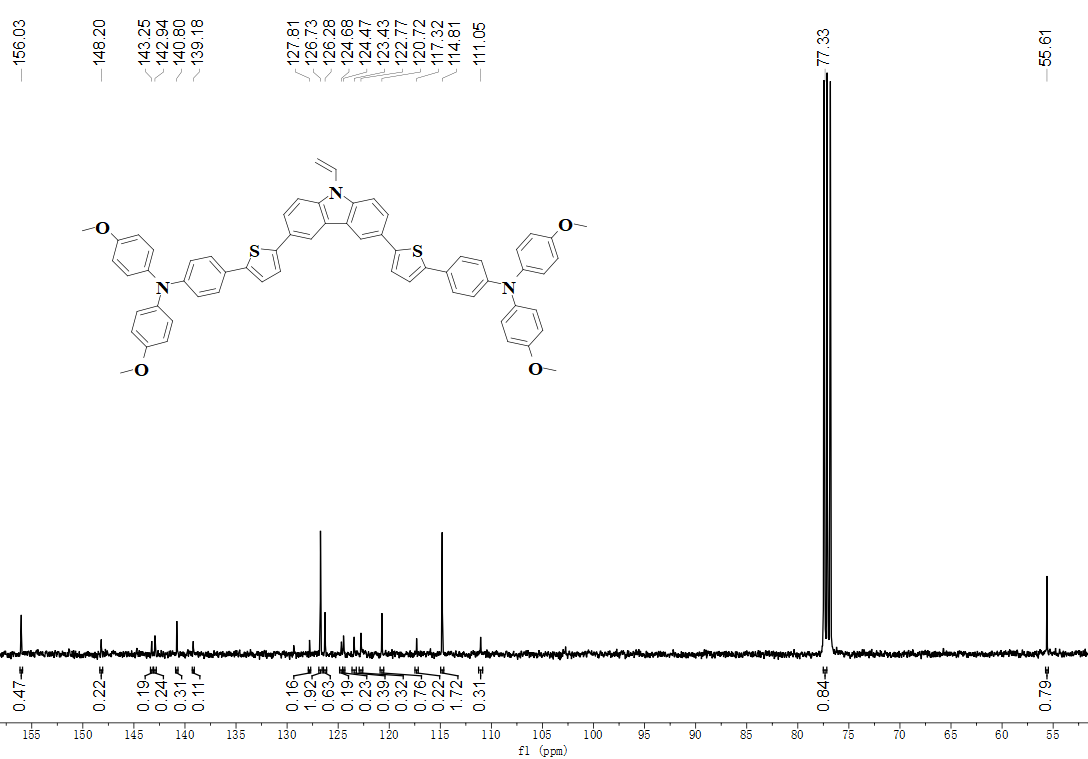


**Figure S30**. ^13^C NMR spectrum of VCz-ThOMeTPA.
